# Supplementary figures and images for: The Escherichia coli Small Protein MntS and Exporter MntP Optimize the Intracellular Concentration of Manganese
Source: PLoS Genet. 2015 Mar 16;11(3):e1004977. doi: 10.1371/journal.pgen.1004977 (PMC4361602; doi:10.1371/journal.pgen.1004977)

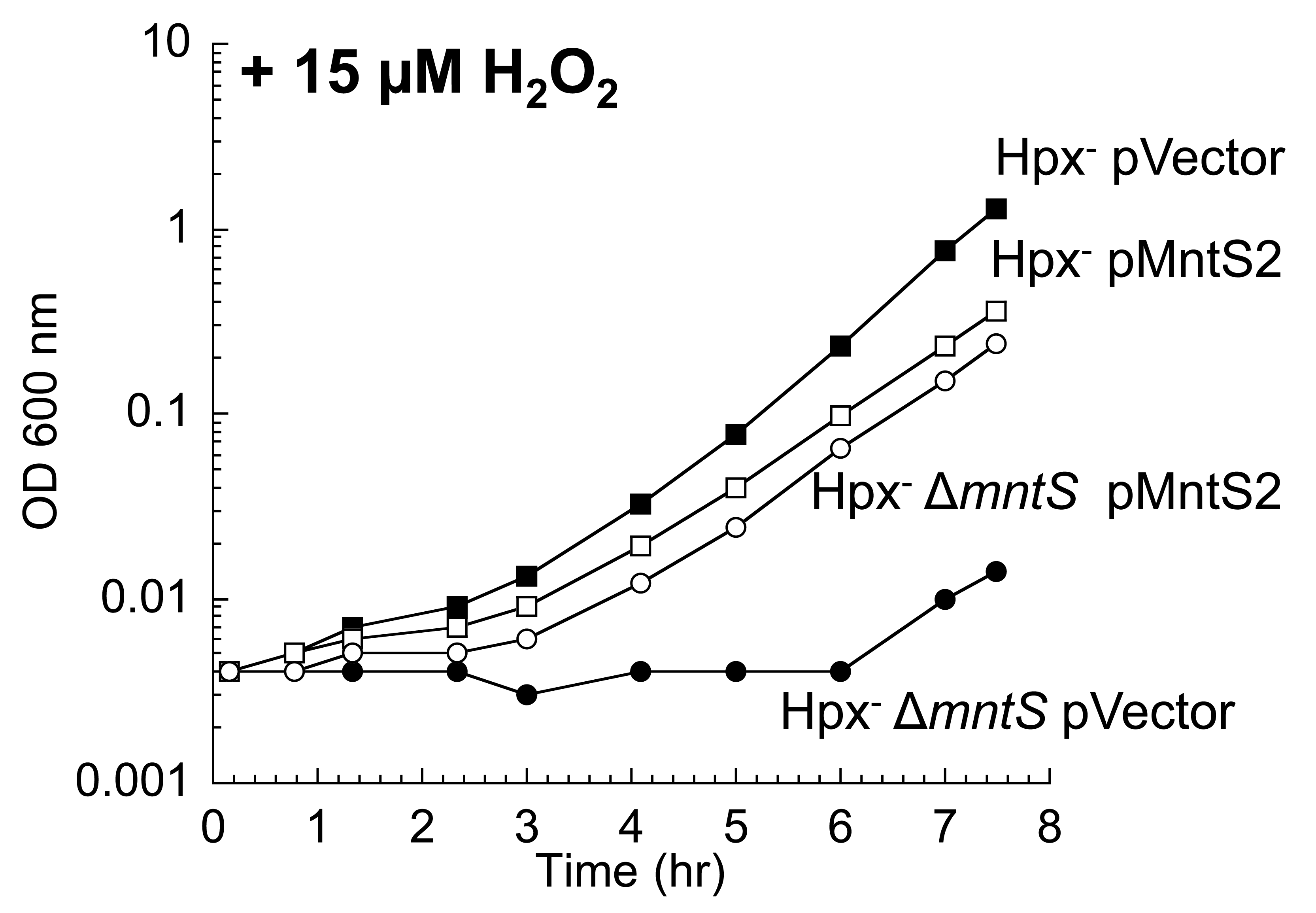

Supplement: S1 Fig — Cells were pre-cultured in anaerobic M9 glucose/casamino acids medium and then diluted at time zero into the same aerobic medium containing 15 μM H2O2. Strains used were LC106 (Hpx-) and JEM1177 (Hpx- ΔmntS) expressing pMntS2 (pLW131, mntS under its own promoter). The data are representative of at least three independent experiments. (TIF) [file pgen.1004977.s001.tif]

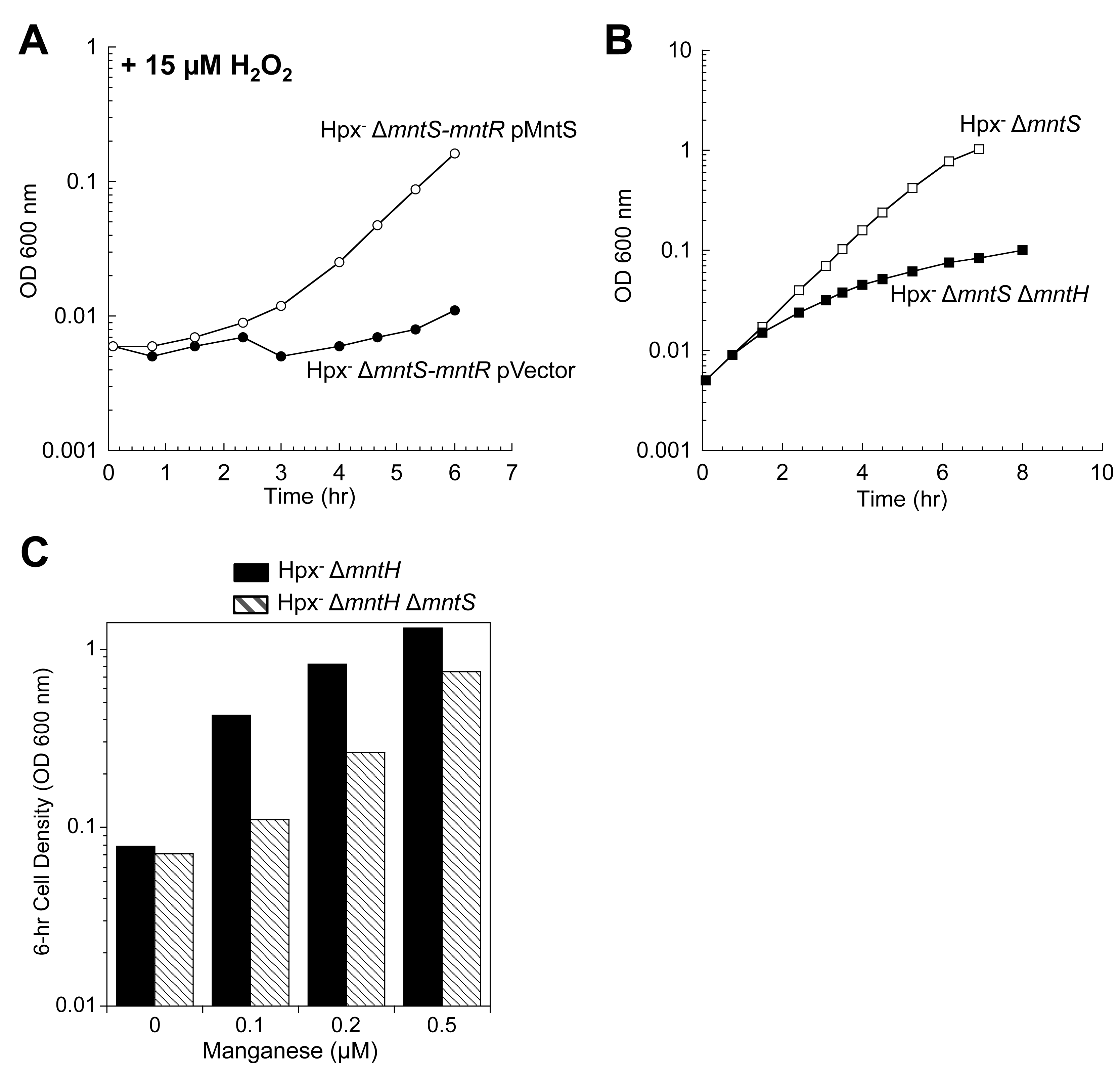

Supplement: S2 Fig — Cells were pre-cultured in anaerobic M9 glucose/casamino acids medium and then diluted at time zero into the same aerobic medium with or without 15 μM H2O2. The data are representative of at least three independent experiments. A. MntS functions in the absence of mntR. Strains used were JEM1216 [Hpx- Δ(mntS-mntR)] carrying an empty vector (pACYC184) or pMntS2 (pLW131, mntS under its own promoter). B. MntH functions in the absence of mntS. Strains were JEM1177 (Hpx- ΔmntS) and JEM1227 (Hpx- ΔmntS ΔmntH). C. MntS functions in the absence of mntH. OD600 of strains AA30 (Hpx- ΔmntH) and JEM1227 (Hpx- ΔmntH ΔmntS) grown in the presence of increasing concentrations of MnCl2 for 6 hr. (TIF) [file pgen.1004977.s002.tif]

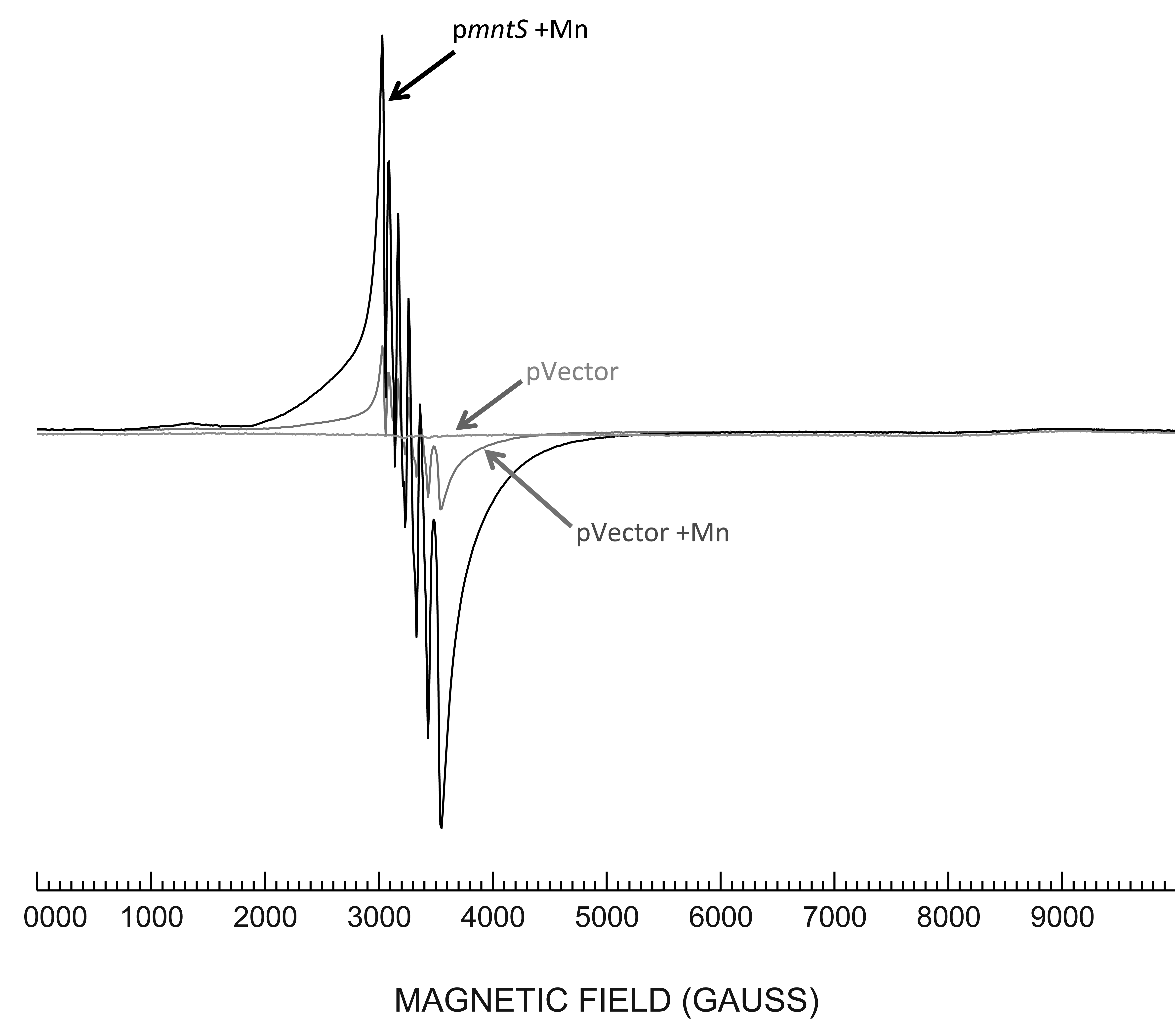

Supplement: S3 Fig — Cultures were grown with 500 μM manganese in LB medium. Peaks represent Mn2+. EPR peak heights varied with point of harvest and do not provide a precise comparison of Mn content. Temperature = 30 K, modulation = 5 g, power = 2 mW. (TIF) [file pgen.1004977.s003.tif]

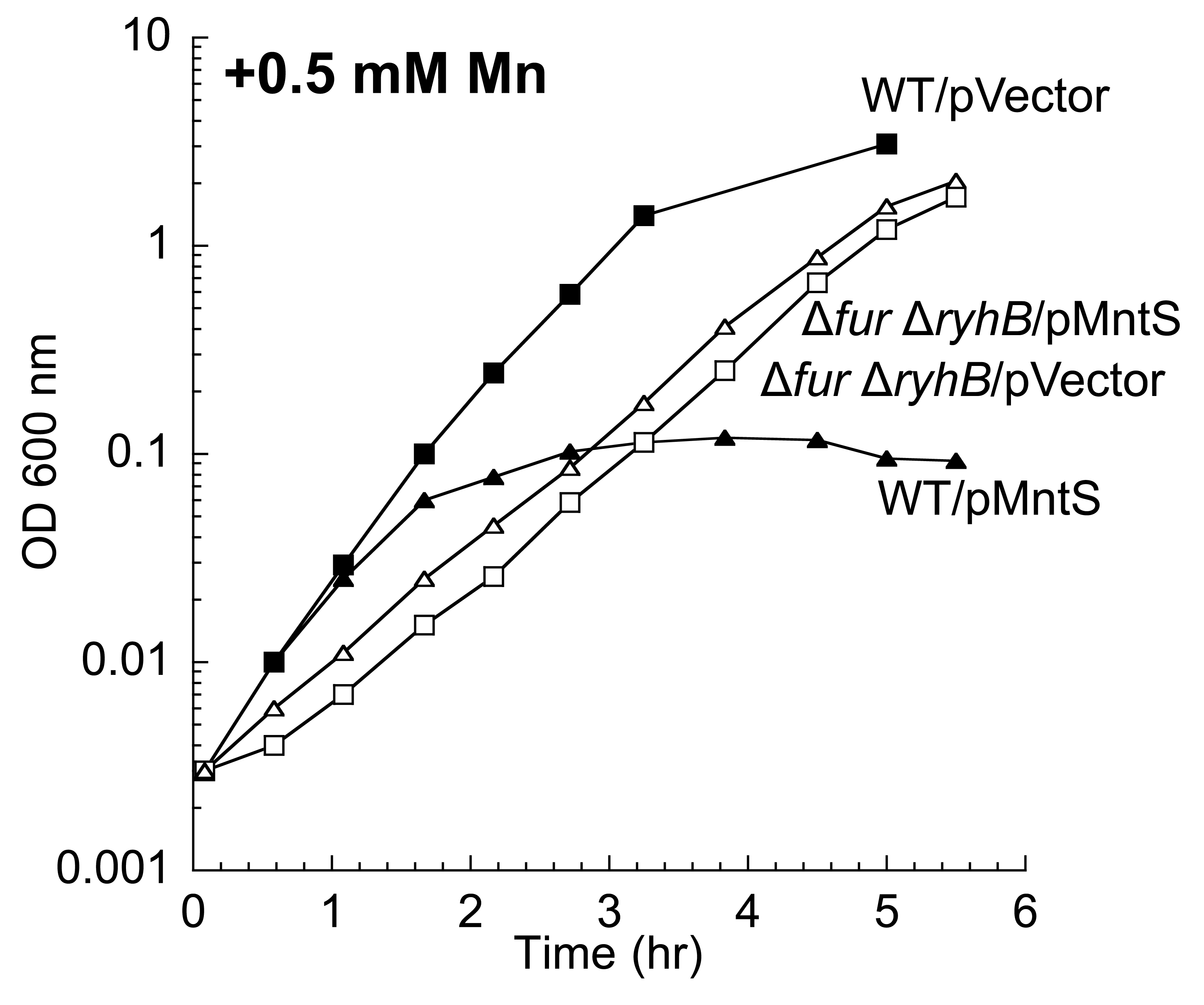

Supplement: S4 Fig — Cells were pre-cultured in aerobic LB medium and then diluted at time zero into fresh LB/arabinose medium with 0.5 mM MnCl2. Strains were JEM1542/JEM1536 (ΔryhB) harboring empty vector (pBAD24) or pMntS (pLW112, mntS driven by the araBAD promoter) and JEM1538/JEM1540 (Δfur ΔryhB) harboring empty vector or pMntS. The data are representative of at least three independent experiments. (TIF) [file pgen.1004977.s004.tif]

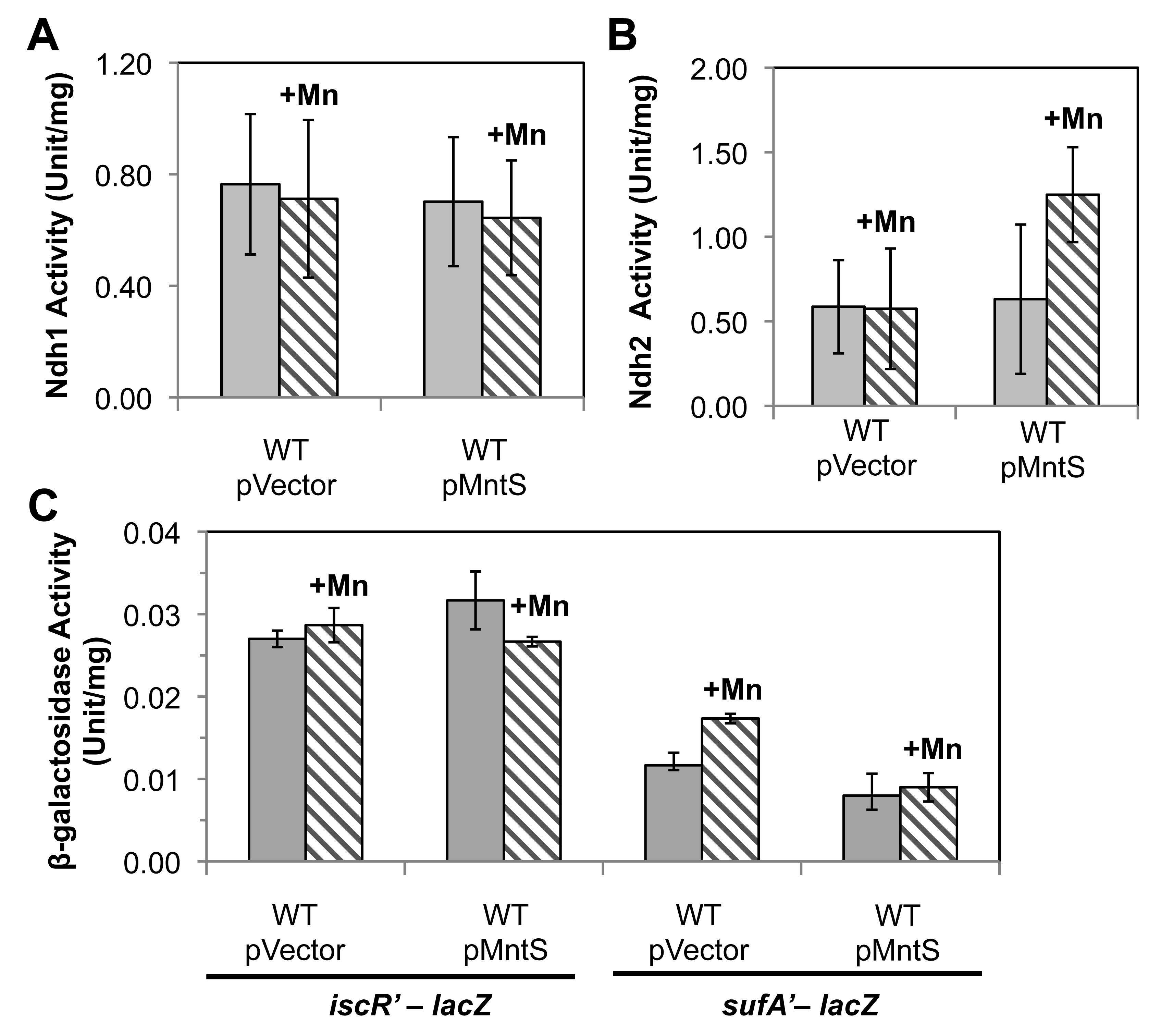

Supplement: S5 Fig — Cells were grown in anaerobic LB/arabinose medium with or without 0.5 mM MnCl2 and aerated for 2.5 hr before harvesting. Data represent the mean of three independent cultures. A, B. Levels of NADH dehydrogenase 1 (an iron-sulfur enzyme) and NADH dehydrogenase 2 (an iron-free enzyme) are not diminished during manganese intoxication. Strains were OD502 (Δsuf) harboring empty vector (pBAD24) or pMntS (pLW112, mntS driven by the araBAD promoter). C. Transcription of the iscR and sufA genes was not induced during manganese intoxication, indicating that IscR remained in its cluster-containing form. Strains bearing iscR’-lacZ were JEM1474 (WT/pBAD24) and JEM1475 (WT/pLW112). Strains bearing sufA’-lacZ were JEM1476 (WT/pBAD24) and JEM1477 (WT/pLW112). (TIF) [file pgen.1004977.s005.tif]

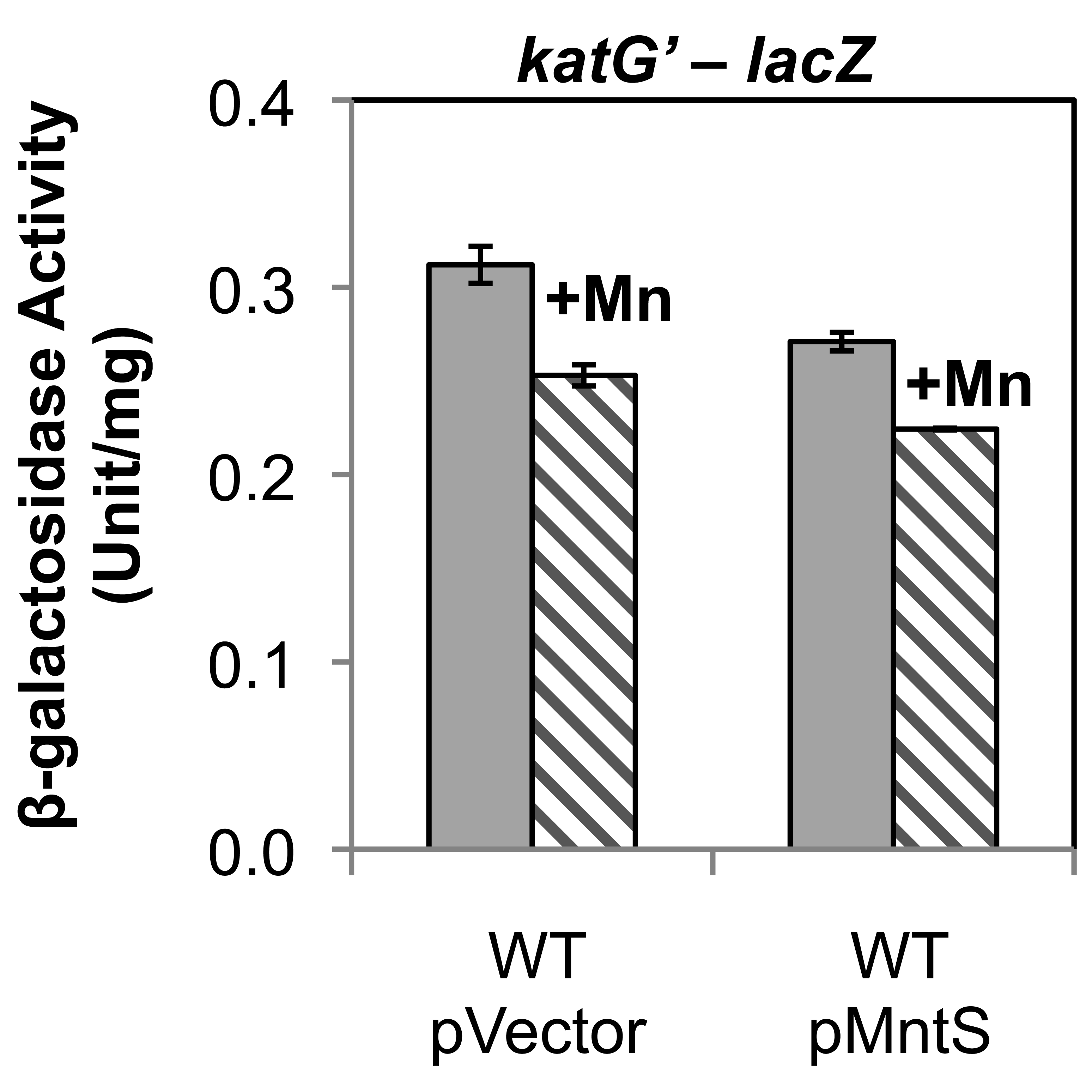

Supplement: S6 Fig — Cells bearing the katG’-lacZ transcriptional fusion were pre-cultured in aerobic LB and then diluted into LB/arabinose medium with or without 0.5 mM MnCl2. Cells were harvested after 2.5 hr treatment. Strains were AL441 harboring empty vector (pBAD24) or pMntS (pLW112). (TIF) [file pgen.1004977.s006.tif]

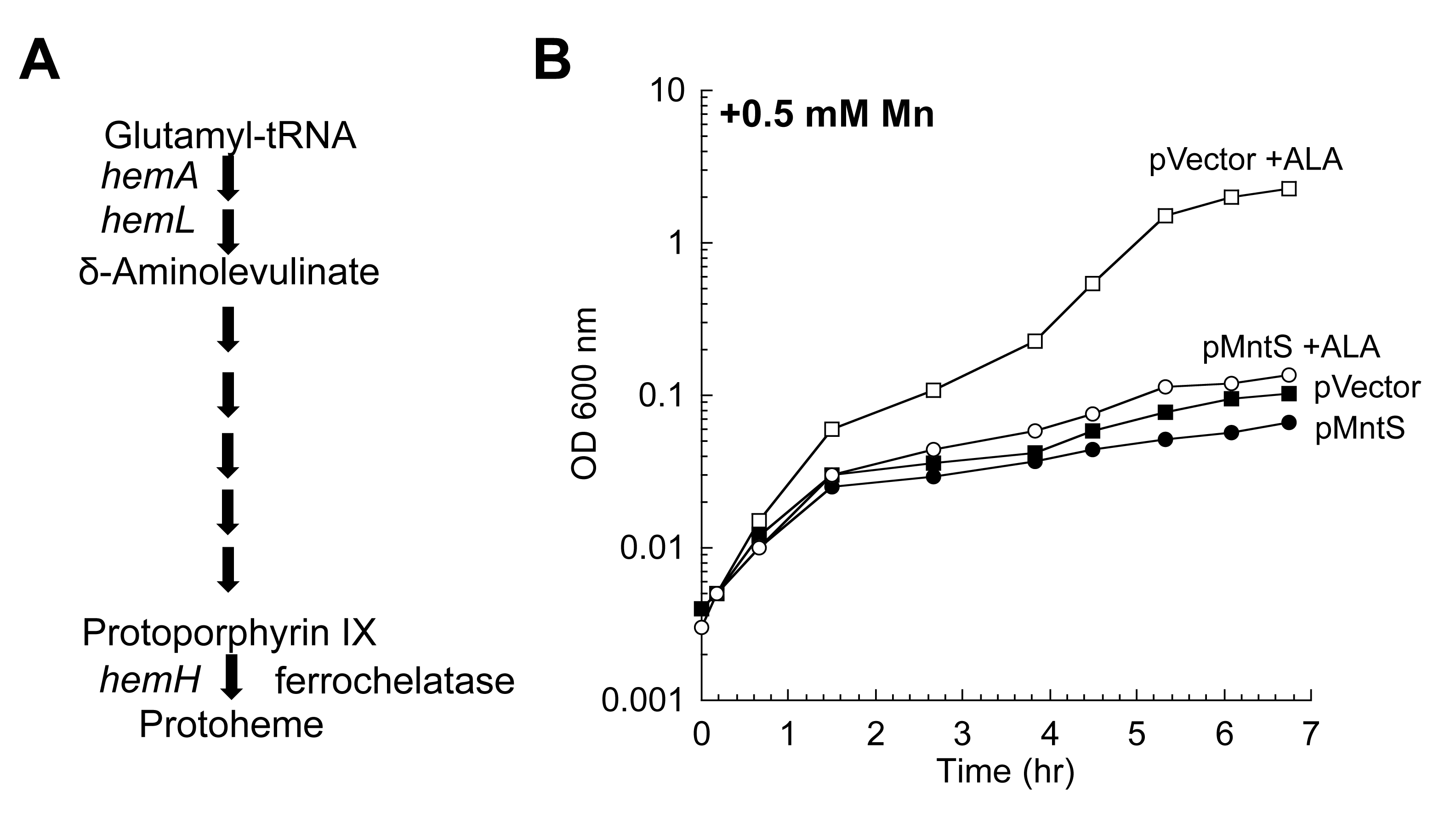

Supplement: S7 Fig — A. The heme biosynthentic pathway. B. Cells lacking hemA were pre-cultured in anaerobic LB medium and then diluted at time zero into fresh aerobic LB/arabinose medium with or without 5-ALA. Strains were SMA1091 (ΔhemA) harboring empty vector (pBAD24) or pMntS (pLW112, mntS driven by the araBAD promoter). The data are representative of at least three independent experiments. (TIF) [file pgen.1004977.s007.tif]

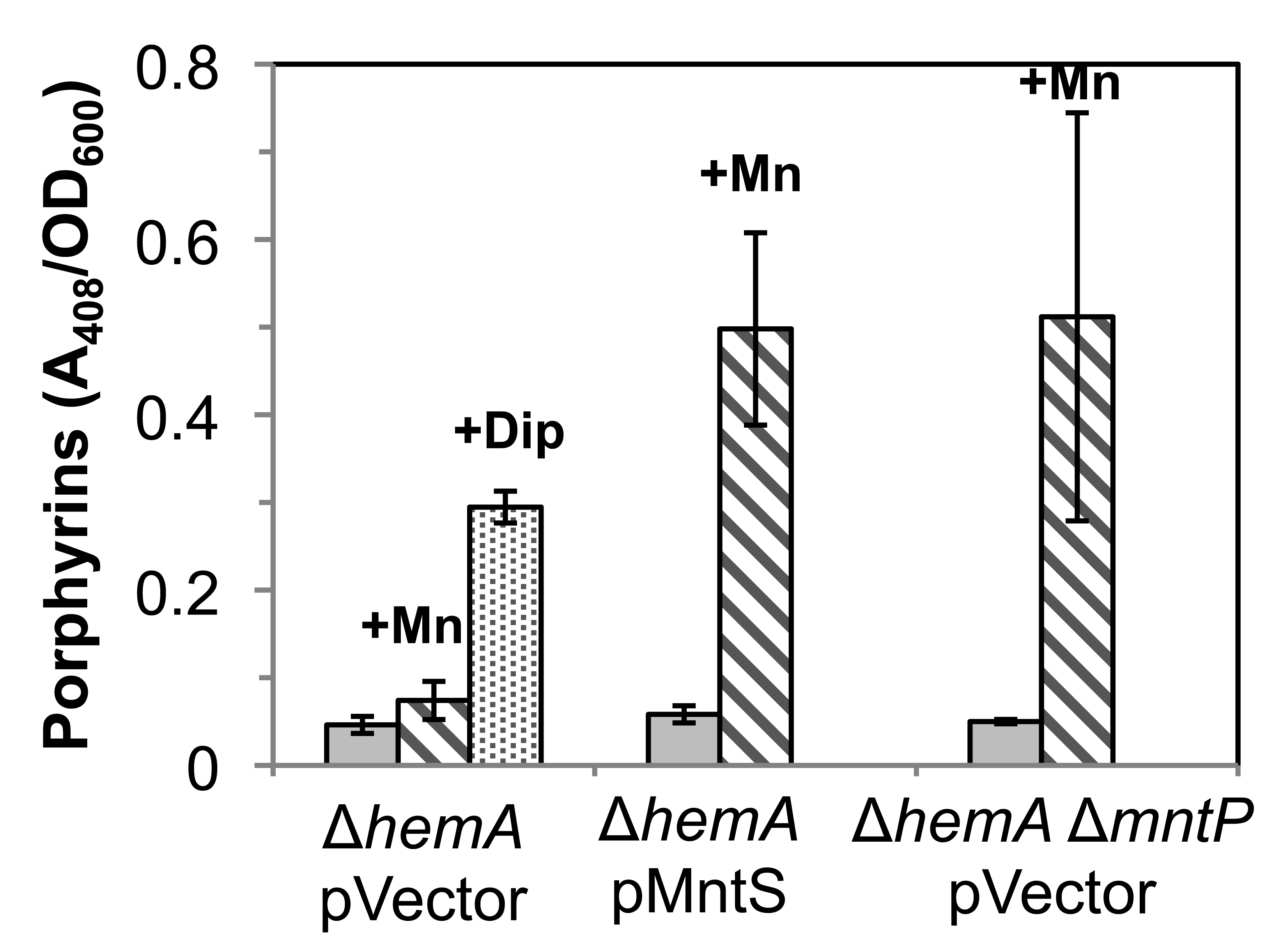

Supplement: S8 Fig — hemA-null mutants were grown in aerobic LB/arabinose medium supplemented with 1 mM 5-ALA. Cells were harvested and intracellular porphyrins were quantified after 2.5 hr treatment with or without 0.5 mM MnCl2 or 100 μM DIP. Data represent the mean of three independent cultures. Strains were JEM1579 (ΔhemA/pBAD24), JEM1580 (ΔhemA/pLW112), JEM1683 (ΔmntP/pBAD24). (TIF) [file pgen.1004977.s008.tif]

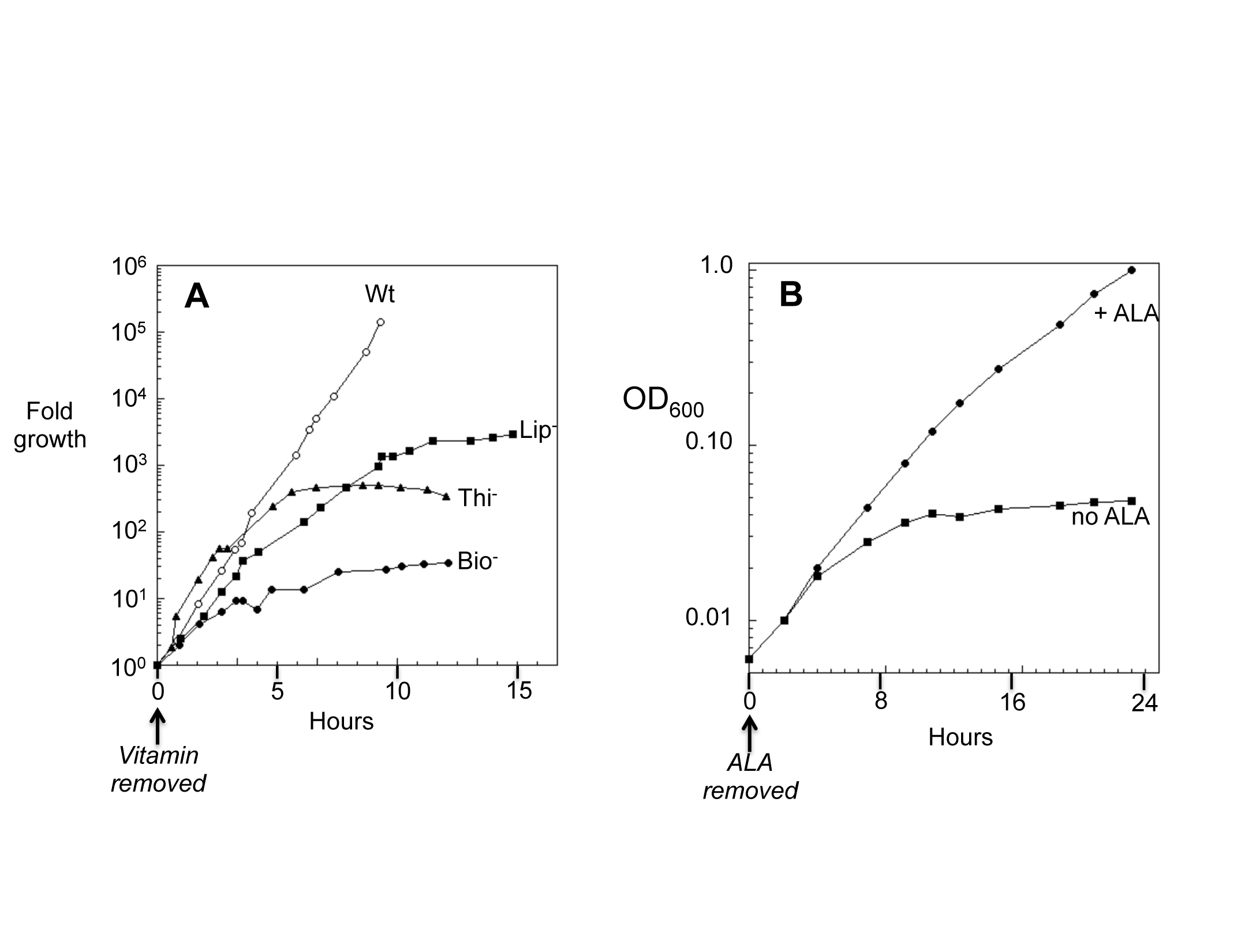

Supplement: S9 Fig — (A) The wild-type strain MG1655 and mutant strains defective in synthesis of thiamine (AB1157), lipoic acid (KER176), and biotin (NRD25) were cultured for 4 generations in minimal A medium supplemented with 0.5 mM of the 20 standard amino acids, plus 5 μg/ml of the required vitamin. At time zero the cells were then centrifuged, washed three times, and suspended in the same media lacking vitamins. Growth was monitored by absorbance. Cells were repeatedly subcultured to maintain densities < 0.3 OD600, and data are presented as the amount of residual growth after removal of the vitamins. (B) A ΔhemA derivative of MG1655 (SMA1139) was cultured > 4 generations in aerobic minimal A medium containing 1% casamino acids as the sole carbon source plus 1 mM 5-aminolevulinic acid (ALA) to enable heme synthesis. The medium was chosen to mimic the effects of LB medium while avoiding the presence of peptides, since ALA is imported through the dipeptide transporter. At time zero the exponentially growing cells were centrifuged, washed three times, and suspended in the same medium +/- ALA, and growth was monitored. (TIF) [file pgen.1004977.s009.tif]

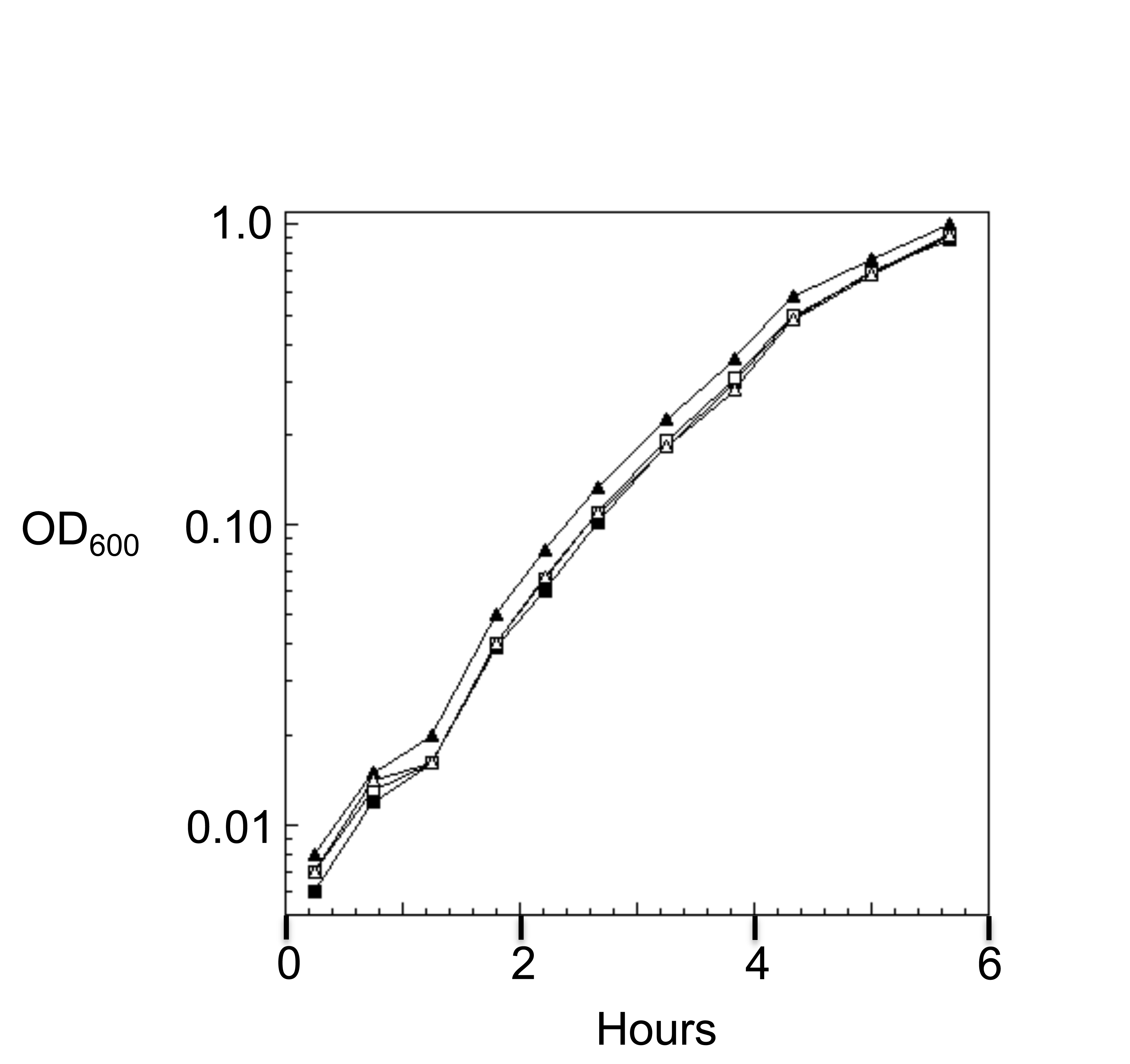

Supplement: S10 Fig — MG1655 strains containing an empty vector (pBAD24; squares) or containing pMntS (pLW112; triangles) were grown exponentially in anoxic LB medium. At time zero cells were diluted into the same medium with no additions (filled symbols) or with 0.5 mM MnCl2 (open symbols), and subsequent growth was monitored. The analogous experiment under aerobic conditions causes complete growth arrest for the Mn-supplemented pMntS strain (Figs 4, 9). (TIF) [file pgen.1004977.s010.tif]

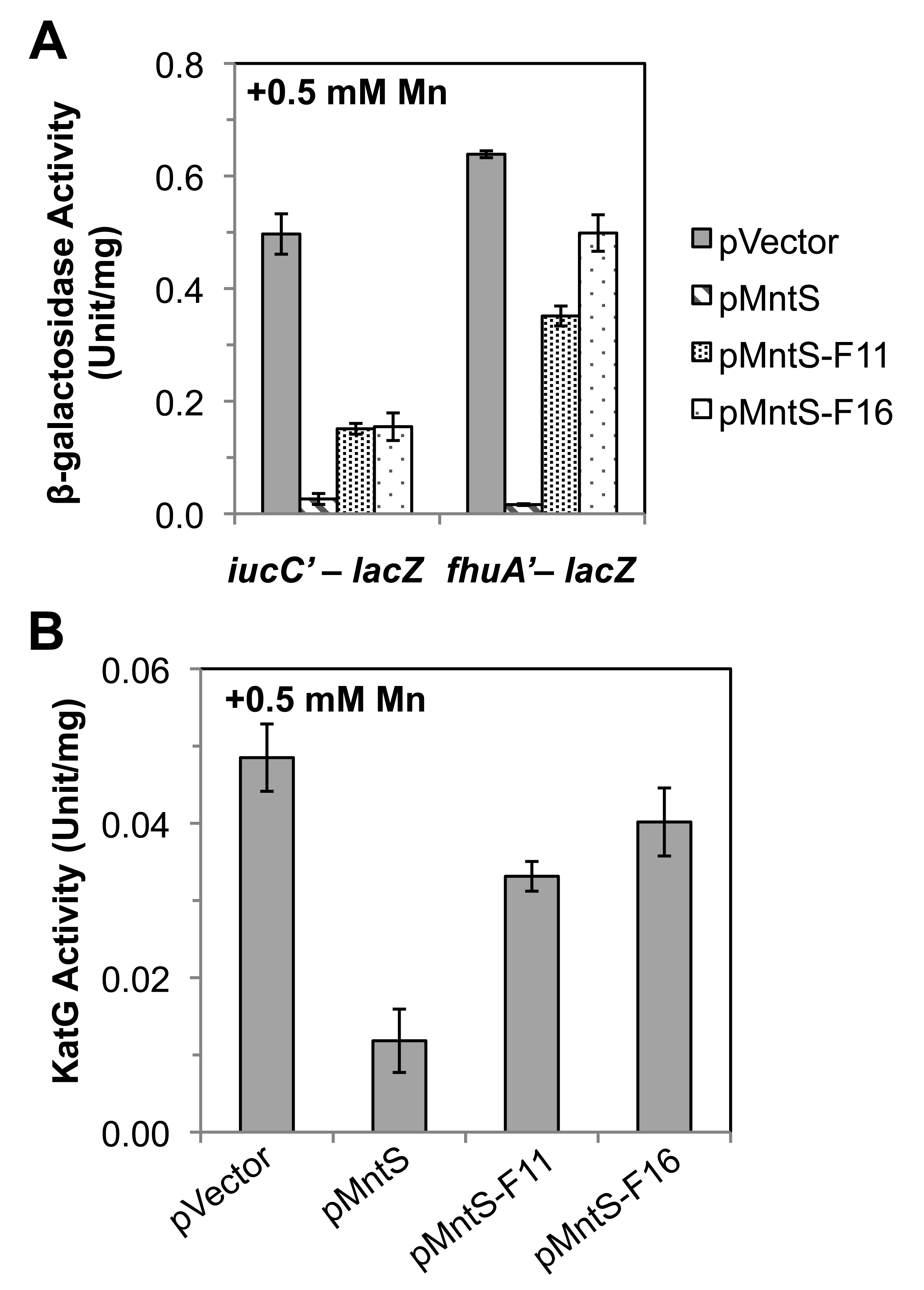

Supplement: S11 Fig — Data represent the mean of three independent cultures. A. Transcription levels of the Fur-regulated genes iucC’-lacZ and fhuA’-lacZ in WT strains (JEM271 and GS45, repectively) harboring the indicated plasmids. B. KatG activity determined from WT (GS45) strains harboring the indicated plasmids. (TIF) [file pgen.1004977.s011.tif]

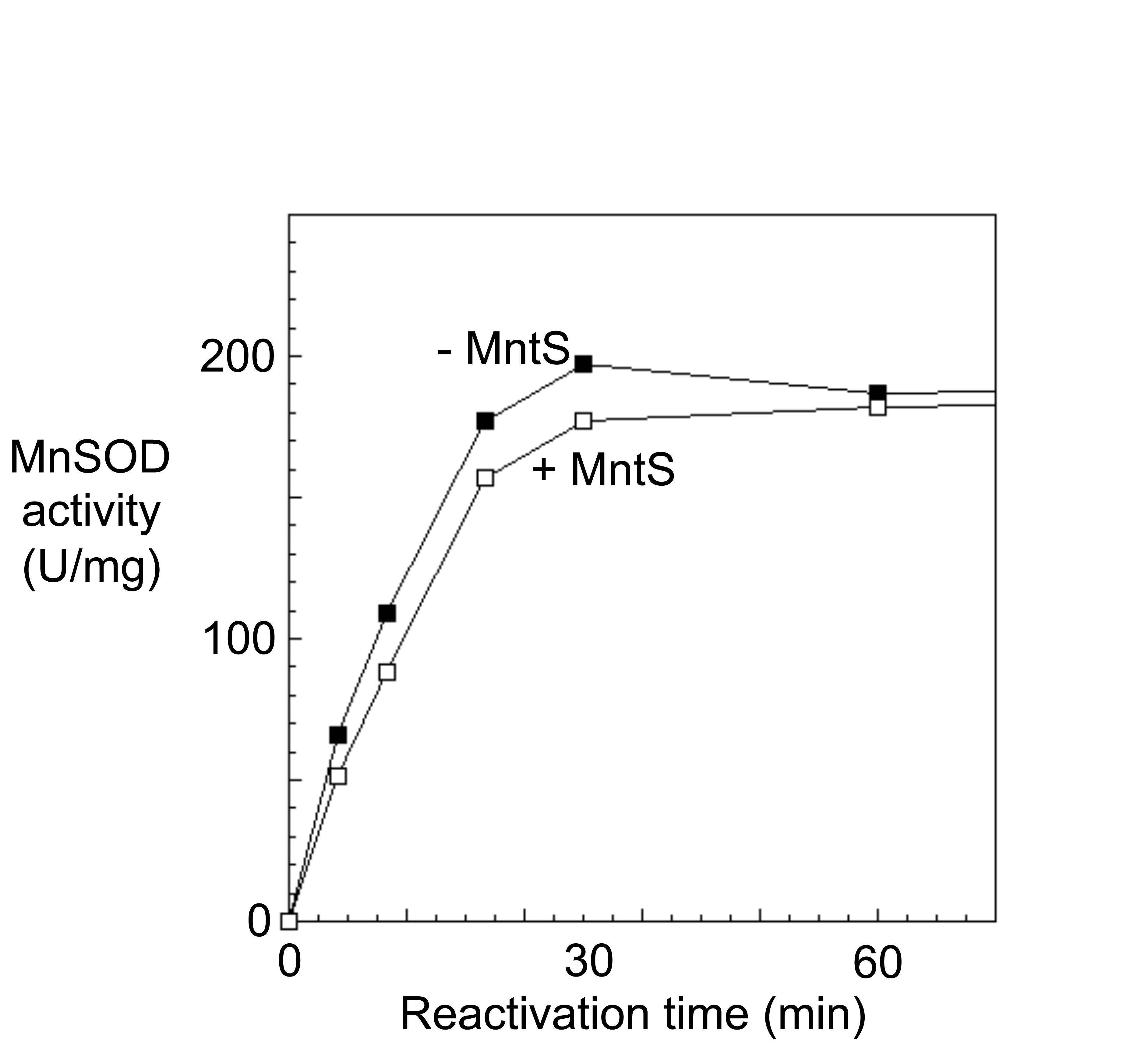

Supplement: S12 Fig — Cell extracts were prepared from the ΔsodB ΔmntS strain JEM1234 containing pDT1–16, which overexpresses sodA. Extract were treated to remove Mn from the MnSOD protein. Remetallation was performed in 37o C pH 7.8 Tris/EDTA buffer through the addition of 200 μM MnCl2, with or without the addition of 0.36 μM purified MntS. The graph has been scaled to emphasize the early time; two- and three-hour time points revealed the same activity as the one-hour time point. (TIF) [file pgen.1004977.s012.tif]

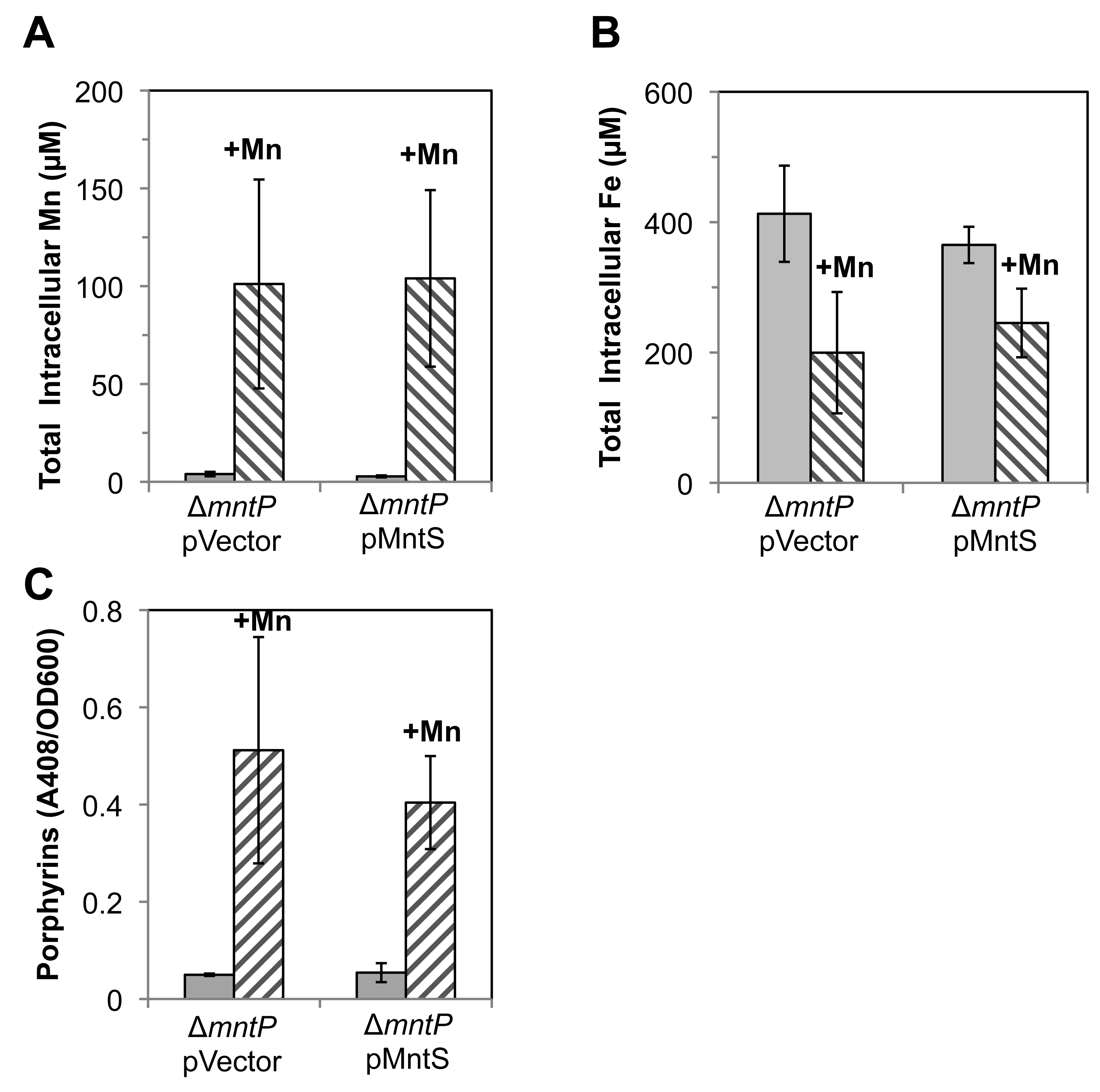

Supplement: S13 Fig — Cells pre-cultured in aerobic LB medium were diluted into fresh LB/arabinose medium with or without 0.5 mM MnCl2 and harvested after 2.5 hr of aerobic growth, followed by ICP-MS analysis (A and B) or porphyrin accumulation (C). Data represent the mean of three independent cultures. Strains were MS025 (ΔmntP) harboring empty vector (pBAD24) or pMntS (pLW112, mntS driven by the araBAD promoter). Note that data represented by empty vector has been reprinted from Fig. 10 to aid data comparison. (TIF) [file pgen.1004977.s013.tif]

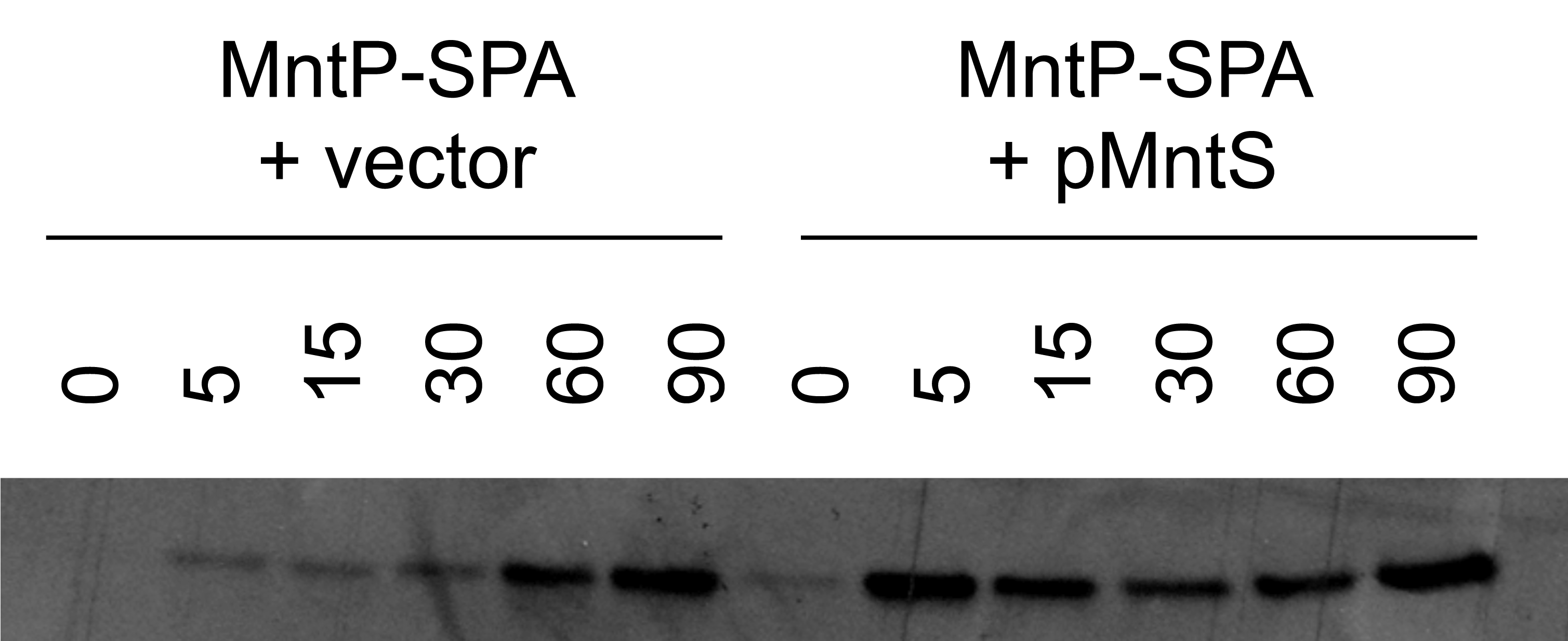

Supplement: S14 Fig — Cultures were grown in M9 glucose medium + ampicillin to 0.2 OD600. Cells were washed and resuspended in arabinose medium for 10 min to induce MntS. They were then washed again and suspended in the original glucose medium supplemented with 10 μM manganese to induce MntP from its native promoter and to reproduce the growth phenotype. At intervals cells were harvested and MntP-SPA content was evaluated by western blot with anti-SPA antibodies. The higher MntP levels in the mntS-overexpressing strains likely results from the increased manganese levels under these conditions (Fig. 5A), since MntP synthesis is induced by manganese [20]. (TIF) [file pgen.1004977.s014.tif]

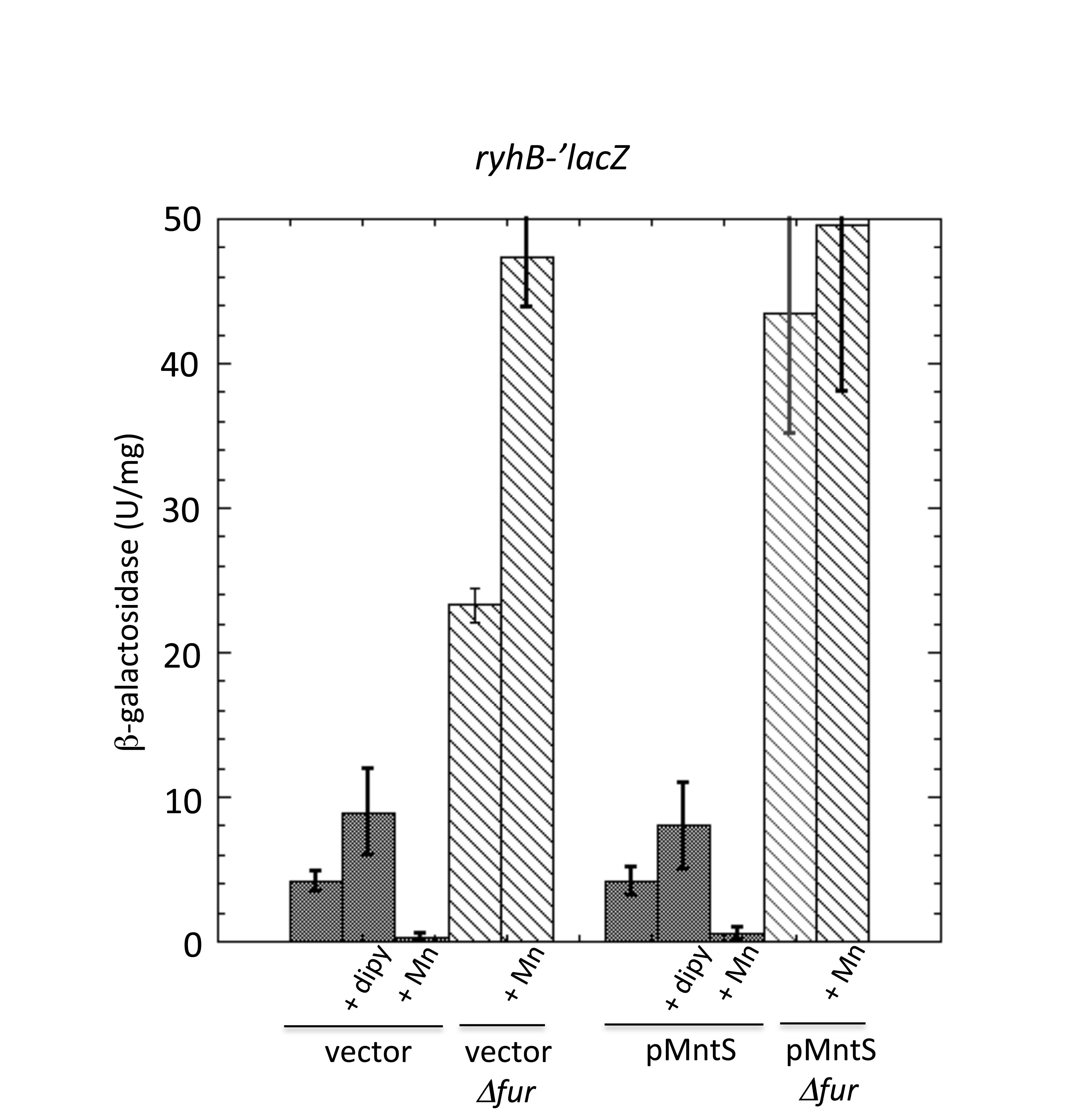

Supplement: S15 Fig — The ryhB-‘lacZ transcriptional fusion strain JEM1500 (with vector), JEM1501 (pMntS), JEM1503 (fur with pBAD vector) and JEM1504 (fur with pMntS) were grown exponentially in aerobic LB/arabinose medium, and ß-galactosidase activity was assayed as a representation of ryhB expression. Where indicated dipyridyl (0.1 mM) was added to moderately restrict iron availability, or an inhibitory dose of manganese (0.5 mM) was added to toxify the pMntS strain. Error bars indicate the standard deviation from 3–4 replicates. (TIF) [file pgen.1004977.s015.tif]
