# Supplementary material for: The Escherichia coli Small Protein MntS and Exporter MntP Optimize the Intracellular Concentration of Manganese
Source: PLoS Genet. 2015 Mar 16;11(3):e1004977. doi: 10.1371/journal.pgen.1004977 (PMC4361602; doi:10.1371/journal.pgen.1004977)
Supplement: S1 Table — (DOCX) [file pgen.1004977.s016.docx]

**Table S1. Strains and plasmids.**

| **Strains** | **Genotype** | **Reference** |
| --- | --- | --- |
| MG1655 | F^-^ wild type | *E. coli* CGSC |
| LC106 | Δ*ahpF::kan* Δ(*katG::*Tn*10*)1 Δ(*katE12::*Tn*10*) | [81] |
| BW25113 | *lacI rrnB* Δ*lacZ hsdK* Δ*araBAD* Δ*rhaBAD* | [66] |
| LSW120 | As MG1655 Δ*mntS_ORF_::kan* | This study |
| MS025 | As MG1655 Δ*mntP::kan* | This study |
| MS023  MS033  LSW195 | As MG1655 Δ(*mntS-mntR*)1*::cat*  As MG1655 *mntP-SPA:kan*  As MG1655 Δ*mntS_RNA_ mntP-SPA:kan* | This study  This study  This study |
| CAG18493 | λ^-^ *rph-1 zbh-29::*Tn*10* | *E. coli* CGSC |
| AA30 | As LC106 Δ*mntH2::cat* | [11] |
| AA99 | As MG1655 Δ*mntH2::cat* | [11] |
| JEM84  JEM510  JEM1186  JEM512  JEM1587 | As MG1655 Δ(*nrdA-nrdB*)*1::cat*  As MG1655 *Δ(lacZ1::cat)1 Δ(nrdHIEF:kan)2::φ(nrdH’-lac^+^)2*  As JEM510 plus *ΔmntS::kan~zbh-29::*Tn*10*  As MG1655 *Δ(lacZ1::cat)1 Δ(nrdHIEF:kan)2::φ(nrdH’-‘lac^+^)2*  As JEM512 plus *Δ(mntS-mntR)::kan~zbh-29::*Tn*10* | [8]  This study  This study  This study  This study |
| JEM609 | As MG1655 Δ*lacZ*1 Δ*tonB1* Δ*feoABC* Δ*zupT::cat* | [8] |
| JEM1136 | As MG1655 Δ*lacZ*1 Δ*tonB1* Δ*feoABC* Δ*zupT* Δ(*nrdA-nrdB*)*1* | This study |
| JEM1253 | As JEM1136 with pACYC184 | This study |
| JEM722 | As MG1655 Δ*lacZ*1 Δ*tonB1* Δ*feoABC* Δ*zupT* Δ(*nrdH-nrdF*)1 Δ(*nrdA-nrdB*)*1::cat~zbf::*Tn*10* | [8] |
| JEM1254 | As JEM1136 with pLW131 | This study |
| JEM1171 | As MG1655 Δ*mntS::kan~zbh-29::*Tn*10* | This study |
| JEM1177 | As LC106 Δ*mntS::kan~zbh-29::*Tn*10* | P1(JEM1171) X LC106 |
| JEM1255 | As JEM1177 with pACYC184 | This study |
| JEM1256 | As JEM1177 with pLW131 | This study |
| JEM1181 | As JEM609 Δ*mntS::kan~zbh-29::*Tn*10* | P1(JEM1171) X JEM609 |
| JEM1202 | As BW25113 Δ*sodB1::cat* | This study |
| JEM1208 | As LC106 Δ*sodB1::cat* | P1(JEM1202) X LC106 |
| JEM1212 | As LC106 Δ*mntS::kan~zjf-29::*Tn*10* Δ*sodB1::cat* | P1(JEM1202) X JEM1177 |
| JEM1222 | As LC106 with pLW131 | This study |
| JEM1214 | As MG1655 Δ(*mntS-mntR*)1*::kan~zbh-29::*Tn*10* | P1(CAG18493) X MS023 |
| JEM1216 | As LC106 Δ(*mntS-mntR*)1*::kan~zbh-29::*Tn*10* | P1(JEM1214) X LC106 |
| JEM1223 | As JEM1216 with pACYC184 | This study |
| JEM1224 | As JEM1216 with pLW131 | This study |
| JEM1227 | As LC106 Δ(*mntS-mntR*)1*::kan~zbh-29::*Tn*10 ΔmntH2::cat* | P1(AA99) X JEM1216 |
| JEM1233 | As MG1655 Δ*sodB1* | P1(JEM1202) X MG1655 |
| JEM1234 | As MG1655 Δ*sodB1* Δ*mntS* | This study |
| JEM1311 | As JEM1234 with pLW131 | This study |
| JEM1244 | As MG1655 with pDT1-16 | This study |
| JEM271  JEM1280  JEM1281 | As MG1655 Δ*lacZ1* pColV  As BL21 (DE3) plus pET21b  As BL21 (DE3) plus pET21b-MntS | This study  This study  This study |
| JEM1369 | As JEM271 with pBAD24 | This study |
| JEM1370 | As JEM271 with pLW112 | This study |
| GS45 | As MG1655 Δ*lacZ1* *attλ*::[pSJ501::f*huA’-lacZ*+]~*cat* | Lab stock |
| **Table S1. (continued)** | | |
| JEM1395 | As GS45 with pBAD24 | This study |
| JEM1396 | As GS45 with pLW112 | This study |
| OD502 | As MG1655 Δ*(sufABCDSE*)*19::kan* | Lab stock |
| JEM1397 | As OD502 with pBAD24 | This study |
| JEM1398 | As OD502 with pLW112 | This study |
| AL441 | As Δ*lacZ1 attλ*::[pSJ501::*katG’*-lacZ+]~*cat* | Lab stock |
| JEM1405 | As AL441 with pBAD24 | This study |
| JEM1406 | As AL441 with pLW112 | This study |
| JEM659 | As BW25113 Δ*hfq1::cat* | This study |
| JEM1408 | As MG1655 Δ*hfq1::cat* | P1(JEM659) X JEM1407 |
| JEM1409 | As JEM1408 with pBAD24 | This study |
| JEM1410 | As JEM1408 with pLW112 | This study |
| JW1316-1 | Δ(*araD-araB*)*567* Δ*lacZ4787*(::*rrnB-3*) λ^-^ Δ*tyrR760::kan rph-1* Δ(*rhaD-rhaB*)*568 hsdR514* | *E. coli* CGSC |
| JEM1413 | As MG1655 Δ*sodB1* Δ*tyrR760::kan* | P1(JW1316-1) X JEM1233 |
| JEM1506 | As MG1655 Δ*sodB1* Δ*mntS ΔtyrR760::kan* | P1(JW1316-1) X JEM1234 |
| JEM913 | As MG1655 Δ*lacZ1* Δ*fur-731* | This study |
| JEM1417 | As JEM913 with pBAD24 | This study |
| JEM1421 | As JEM913 with pLW112 | This study |
| JEM1425 | As JEM913 *attλ*::[pSJ501::*katG’-lacZ*+]~*cat* | P1(AL441) X JEM913 |
| JEM1427 | As JEM1425 with pBAD24 | This study |
| JEM1428 | As JEM1425 with pLW112 | This study |
| JEM1431 | As JEM913 attλ::[pSJ501::katG’-lacZ+] Δ*ryhB1::cat* | This study |
| JEM1447 | As JEM1431 with pBAD24 | This study |
| JEM1448 | As JEM1431 with pLW112 | This study |
| JEM1453 | As MG1655 ΔP*_katG_*:*:tetRA*-23 with pBAD24 | This study |
| JEM1454 | As MG1655 ΔP*_katG_*:*:tetRA*-23 with pLW112 | This study |
| JEM1455 | As JEM913 attλ::[pSJ501::katG’-lacZ+] Δ*mntH2::cat* | This study |
| JEM1457 | As MG1655 ΔP*_katG_*:*:tetRA*-23 Δ*fur-731::kan* | This study |
| JEM1459 | As JEM913 attλ::[pSJ501::katG’-lacZ+] Δ*iscR::cat* | This study |
| JEM981 | As MG1655 Δ*lacZ1* Δ*fur-731::kan* with pColV | This study |
| JEM1463 | As JEM981 with pBAD24 | This study |
| JEM1464 | As JEM981 with pLW112 | This study |
| JEM1465 | As MG1655 Δ*lacZ1* *attλ*::[pSJ501::f*huA’-lacZ*+]~*cat*Δ*fur-731::kan* with pBAD24 | This study |
| JEM1466 | As MG1655 Δ*lacZ1* *attλ*::[pSJ501::f*huA’-lacZ*+]~*cat*Δ*fur-731::kan* with pLW112 | This study |
| SJ169 | As MG1655 Δ*lacZ1* *attλ*::[pSJ501::*iscR’-lacZ*+]~*cat* | Lab stock |
| JEM1474 | As SJ169 with pBAD24 | This study |
| JEM1475 | As SJ169 with pLW112 | This study |
| SJ253 | As MG1655 Δ*lacZ1* *attλ*::[pSJ501::*sufA’-lacZ*+]~*cat* | Lab stock |
| JEM1476 | As SJ253 with pBD24 | This study |
| JEM1477 | As SJ253 with pLW112 | This study |
| **Table S1. (continued)** | | |
| JS248 | As MG1655 Δ*aroB1::cat* | Lab stock |
| JEM1494  JEM1500  JEM1501  JEM1503  JEM1504 | As JEM1234 Δ*aroB1::cat*  As JAB017 with pBAD24  As JAB017 with pLW112  As JAB107 *Δfur-731::kan* plus pBAD24  As JAB107 *Δfur-731::kan* plus pLW112 | P1(JS248) X JEM1234  This study  This study  This study  This study |
| JEM1522 | As JEM1233 Δ*mntP::kan* | P1(MS025) X JEM1233 |
| JEM1524 | As JEM1234 Δ*mntP::kan* | P1(MS025) X JEM1234 |
| JEM1538 | As MG1655 Δ*lacZ1* Δ*fur-731* Δ*ryhB::cat* with pBAD24 | This study |
| JEM1540 | As MG1655 Δ*lacZ1* Δ*fur-731* Δ*ryhB::cat* with pLW112 | This study |
| SMA1091 | As MG1655 Δ*lacZ1* Δ*hemA::kan attλ*::[pSJ501::*hemA’-lacZ*+] | Lab stock |
| JEM1579 | As SMA1091 with pBAD24 | This study |
| JEM1580 | As SMA1091 with pLW112 | This study |
| AA171 | As MG1655 Δ*lacZ1 attλ*::[pSJ501::*mntH’-lacZ*+]~*cat* | [8] |
| JEM1609 | As JEM913 *attλ*::[pSJ501:*:mntH’-lacZ*+]~*cat* | P1(AA171) X JEM913 |
| JEM1647 | As JEM913 *attλ*::[pSJ501:*:mntH’-lacZ*+] | This study |
| JEM1651 | As JEM1647 Δ*mntH2::cat* | P1(AA99) X JEM1647 |
| AA46 | As MG1655 Δ*mntR1::cat* | Lab stock |
| JEM1667 | As JEM1647 Δ*mntR1::cat* | P1(AA46) X JEM1647 |
| JEM1611 | As JEM913 Δ*mntS* *attλ*::[pSJ501:*:mntH’-lacZ*+]~*cat* | This study |
| JEM1649 | As JEM913 Δ*mntS* *attλ*::[pSJ501:*:mntH’-lacZ*+] | This study |
| JEM1653 | As JEM1649 Δ*mntH2::cat* | P1(AA99) X JEM1649 |
| JEM1663 | As BW25113 Δ*mntP2::cat* | This study |
| JEM1682 | As SMA1091 Δ*mntP2::cat* | P1(JEM1663) X SMA1091 |
| JEM1683 | As JEM1682 with pBAD24 | This study |
| JEM1684 | As JEM1682 with pLW112 | This study |
| JEM1715 | As MG1655 Δ*mntS* Δ*mntP::kan* | This study |
| JEM1719 | As JEM271 Δ*mntP::kan* | P1(MS025) X JEM71 |
| JEM1713 | As MG1655 Δ*lacZ1* *attλ*::[pSJ501::f*huA’-lacZ*+] | This study |
| JEM1720 | As JEM1713 Δ*mntP::kan* | P1(MS025) X JEM1713 |
| JEM1714 | As MG1655 Δ*lacZ1* *attλ*::[pSJ501::f*huA’-lacZ*+] Δ*fur-731* | This study |
| JEM1722 | As JEM1714 Δ*mntP::kan* | P1(MS025) X JEM1714 |
| JEM1718 | As MG1655 Δ*lacZ1* Δ*fur-731* with pColV | This study |
| JEM1724 | As JEM1718 Δ*mntP::kan* | P1(MS025) X JEM1718 |
| JEM1235 | As MG1655 Δ*sodB1* Δ*mntH2::cat* | This study |
| JEM1237 | As MG1655 Δ*sodB1* Δ*mntS* Δ*mntH2::cat* | This study |
| MG1655/pBAD24 | As MG1655 with pBAD24 | This study |
| MG1655/pLW112 | As MG1655 with pLW112 | This study |
| JEM1726  SMA1139  AB1167  KER176  NRD25  JAB017 | As MS025 with pBAD24  As MG1655 *ΔhemA1::cat*  F^-^ *thr-1 leuB6 proA2 his-4 thi-1 argE2 lacY1 galK2 rpsL supE44 ara-14 xyl-15 mtl-1 tsx-33*  W3110 *rpsL lipA::*Tn*1000kan*  F^-^ *araD139 Δ(ara-leu7696 galE15 galK16 Δ(lac)X74 rpsL (Str^R^) hsdR2 (r_K_-m_K_+) mcrA mcrB1 Δ(bioA-bioD)::cm*  *ryhB’-lacZ ΔlacZ X174* | This study  [82]  John Cronan  John Cronan  Eric Massé |
| **Table S1. (continued)** | | |
| JEM1727 | As MS025 with pLW112 | This study |
| JEM1571 | As MG1655 with pJEM67 | This study |
| JEM1575 | As MG1655 with pJEM68 | This study |
| JEM1603 | As JEM271 with pJEM67 | This study |
| JEM1604 | As JEM271 with pJEM68 | This study |
| JEM1290 | As MG1655 with pMS17 | This study |
| JEM1291 | As MG1655 with pMS18 | This study |
| JEM1293 | As MG1655 with pMS20 | This study |
| JEM1294 | As MG1655 with pMS21 | This study |
| JEM1295 | As MG1655 with pLW125 | This study |
| JEM1335 | As MG1655 with pLW133 | This study |
| JEM1336 | As MG1655 with pLW134 | This study |
| JEM1337 | As MG1655 with pLW135 | This study |
| JEM1338 | As MG1655 with pLW136 | This study |
|  |  |  |
|  |  |  |
|  |  |  |
| **Plasmid** | **Relevant characteristics** | **Reference** |
| pBAD24 | Amp^R^ ColE1 with arabinose-responsive promoter | Lab stock |
| pLW112 | pBAD24 containing *mntS* ORF and its own Shine-Dalgarno | [20] |
| pJEM67 | pBAD24 containing *mntS*-(Phe11 +1 frameshift) | This study |
| pJEM68 | pBAD24 containing *mntS*-(Phe16 +1 frameshift) | This study |
| pMS17 | pBAD24 containing *mntS*-(E3A) | This study |
| pMS18 | pBAD24 containing *mntS*-(C7A) | This study |
| pMS20 | pBAD24 containing *mntS*-(C27A) | This study |
| pMS21 | pBAD24 containing *mntS*-(D28A) | This study |
| pLW125 | pBAD24 containing *mntS*-(H13A) | This study |
| pLW133 | pBAD24 containing *mntS*-(E3A/C7A/D28A) | This study |
| pLW134 | pBAD24 containing *mntS*-(E3A/C27A/D28A) | This study |
| pLW135 | pBAD24 containing *mntS*-(C7A/C27A/D28A) | This study |
| pLW136 | pBAD24 containing *mntS*-(E3A/C7A/C27A) | This study |
| pACYC184 | Tet^R^ Cam^R^ | [83] |
| pLW131 | pACYC184 containing *rybA* (includes *mntS* ORF) | This study |
| pKD3 | *bla* FRT *cat* FRT PS1 PS2 oriR6K | [66] |
| pKD46 | *bla* P_BAD_ *gam bet exo* pSC101 oriTS | [66] |
| pCP20 | *bla cat cI857* λP_R_ *flp* pSC101 oriTS | [84] |
| pSJ501 | pAH125 derivative with *cat* flanked by *flp* sites | Lab stock |
| pDT1-16 | pBR322 containing *sodA* under *tac* promoter, Amp^R^ | Lab stock |
|  |  |  |

Additional references for Table S1.

81. Seaver LC, Imlay JA (2004) Are respiratory enzymes the primary sources of intracellular hydrogen peroxide? J Biol Chem 279: 48742-48750.

82. Bachmann, BJ (1996) Derivations and genotypes of some mutant derivatives of *Escherichia coli* K-12. In: *Escherichia coli* and *Salmonella*. FC Neidhardt *et al*., editors. ASM Press, Washington, D.C.

83. Kullik I, Toledano MB, Tartaglia LA, Storz G (1995) Mutational analysis of the redox-sensitive transcriptional regulator OxyR: regions important for oxidation and transcriptional activation. J Bacteriol 177: 1275-1284.

84. Cherepanov PP, Wackernagel W (1995) Gene disruption in *Escherichia coli*: Tc^R^ and Km^R^ cassettes with the option of Flp-catalyzed excision of the antibiotic-resistance determinant. Gene 158: 9-14.
