# Supplementary material for: The Escherichia coli Small Protein MntS and Exporter MntP Optimize the Intracellular Concentration of Manganese
Source: PLoS Genet. 2015 Mar 16;11(3):e1004977. doi: 10.1371/journal.pgen.1004977 (PMC4361602; doi:10.1371/journal.pgen.1004977)
Supplement: S2 Table — (DOCX) [file pgen.1004977.s017.docx]

| **Table S2. Primers used for site-directed mutagenesis.** | |
| --- | --- |
| **Primer** | **Sequence** |
| MntS-Phe11 +1frameshift | 5′-TGAATGAGTTCAAGAGGTGTATGCGCGTGTaTTAGTCATTCTC  CCTTTAAAGTACGGTTA-3′ |
| MntS-Phe16 +1frameshift | 5′-AGGTGTATGCGCGTGTTTAGTCATTCTCCCTaTTAAAGTACGGTT  AATGCTGCTCTCTAT-3′ |
| MntS-Glu3Ala | 5′-AGGAGGTCTTATGAATGcGTTCAAGAGGTGTATGCGCGTGTTTA  GTCATTCTCCCTTTAA-3′ |
| MntS-Cys7Ala | 5′-AGGAGGTCTTATGAATGAGTTCAAGAGGgcTATGCGCGTGTTTA  GTCATTCTCCCTTTAA-3′ |
| MntS-Cys27Ala | 5′-AAGTACGGTTAATGCTGCTCTCTATGTTGgcCGATATGGTCAACA  ACAAACCGCAGCAAG-3′ |
| MntS-Asp28Ala | 5′-GTACGGTTAATGCTGCTCTCTATGTTGTGCGcTATGGTCAACA  ACAAACCGCAGCAAGAT-3′ |
| MntS-His13Ala | 5′-GTTCAAGAGGTGTATGCGCGTGTTTAGTgcTTCTCCCTTTAAAGT  ACGGTTAATGCTG-3′ |
| MntS-E3A/C7A/D28A | 5′-AGGAGGTCTTATGAATGcGTTCAAGAGGgcTATGCGCGTGTTT  AGTCATTCTCCCTTTAA-3′ |
| MntS-E3A/C27A/D28A | 5′-AAGTACGGTAATGCTGCTCTCTATGTTGgcCGCTATGGTCAACA  ACAAACCGCAGCAAG-3′ |
| MntS-C7A/C27A/D28A | 5′-AAGTACGGTTAATGCTGCTCTCTATGTTGgcCGcTATGGTCAACA  ACAAACCGCAGCAAG-3′ |
| MntS-E3A/C7A/C27A | 5′-AGGAGGTCTTATGAATGcGTTCAAGAGGgcTATGCGCGTGTTTA  GTCATTCTCCCTTTAA-3′ |
