# Supplementary material for: The Escherichia coli Small Protein MntS and Exporter MntP Optimize the Intracellular Concentration of Manganese
Source: PLoS Genet. 2015 Mar 16;11(3):e1004977. doi: 10.1371/journal.pgen.1004977 (PMC4361602; doi:10.1371/journal.pgen.1004977)
Supplement: S3 Table — Non-K-12 strains additionally express heme-containing soluble cytochrome b562 (CybC) and ferrous iron transporter (EfeUOB), both of which are cryptic in K-12 strains. E. coli K-12 synthesizes two proteins with siroheme cofactors: sulfite reductase and soluble nitrite reductase (NirB). Ferrochelatase is not involved in siroheme synthesis. (DOCX) [file pgen.1004977.s018.docx]

**Table S3. Heme proteins of *Escherichia coli* K-12.**

| **Enzyme** | **Gene(s)** | **Role** |
| --- | --- | --- |
| catalase G | *katG* | H_2_O_2_ degradation, exponential phase |
| catalase E | *katE* | H_2_O_2_ degradation, stationary phase |
| cytochrome bo oxidase | *cyoABCD* | respiration |
| cytochrome bd oxidase I | *cydAB* | respiration |
| cytochrome bd oxidase II | *appCD* | respiration |
| formate dehydrogenase-O | *fdoGHI* | formate oxidation |
| succinate dehydrogenase | *sdhCDAB* | succinate oxidation |
| bacterioferritin | *bfr* | iron storage |
| c-di-GMP cyclase | *dosCP* | regulation of c-di-GMP |
| flavohemoglobin | *hmp* | nitric oxide scavenging |
| cytochrome c peroxidase | *yhjA* | unknown (anoxic) |
| nitrite reductase | *nrfABCD* | anaerobic respiration (anoxic) |
| nitrate reductase | *napABCGH* | anaerobic respiration (anoxic) |
