# Supplementary material for: Correction: The Escherichia coli Small Protein MntS and Exporter MntP Optimize the Intracellular Concentration of Manganese
Source: PLoS Genet. 2015 Jun 15;11(6):e1005322. doi: 10.1371/journal.pgen.1005322 (PMC4468257; doi:10.1371/journal.pgen.1005322)
Supplement: S1 File — (PDF) [file pgen.1005322.s001.pdf]

PLOS | GENETICS

RESEARCH ARTICLE

The *Escherichia coli* Small Protein MntS and Exporter MntP Optimize the Intracellular Concentration of Manganese

Julia E. Martin[1], Lauren S. Waters[2]*, Gisela Storz[3], James A. Imlay[1]*

1 Department of Microbiology, University of Illinois, Urbana, Illinois, United States of America, **2** Department of Chemistry, University of Wisconsin Oshkosh, Oshkosh, Wisconsin, United States of America, **3** Cell Biology and Metabolism Program, Eunice Kennedy Shriver National Institute of Child Health and Human Development, Bethesda, Maryland, United States of America

* watersl@uwosh.edu (LSW); jimlay@illinois.edu (JAI)

CrossMark
click for updates

OPEN ACCESS

Citation: Martin JE, Waters LS, Storz G, Imlay JA (2015) The *Escherichia coli* Small Protein MntS and Exporter MntP Optimize the Intracellular Concentration of Manganese. PLoS Genet 11(3): e1004977. doi:10.1371/journal.pgen.1004977

Received: June 8, 2014

Accepted: December 30, 2014

Published: March 16, 2015

Copyright: This is an open access article, free of all copyright, and may be freely reproduced, distributed, transmitted, modified, built upon, or otherwise used by anyone for any lawful purpose. The work is made available under the Creative Commons CC0 public domain dedication.

Data Availability Statement: All relevant data are within the paper and its Supporting Information files.

Funding: This work was funded by grants GM101012 from the National Institutes of Health (www.nih.gov) (JAI JEM); 5T32-GM070421 from the National Institutes of Health (JEM); and The Intramural Research Program of the National Institutes of Health (LSW GS). The funders had no role in study design, data collection and analysis, decision to publish, or preparation of the manuscript.

Competing Interests: The authors have declared that no competing interests exist.

Abstract

Escherichia coli does not routinely import manganese, but it will do so when iron is unavailable, so that manganese can substitute for iron as an enzyme cofactor. When intracellular manganese levels are low, the cell induces the MntH manganese importer plus MntS, a small protein of unknown function; when manganese levels are high, the cell induces the MntP manganese exporter and reduces expression of MntH and MntS. The role of MntS has not been clear. Previous work showed that forced MntS synthesis under manganese-rich conditions caused bacteriostasis. Here we find that when manganese is scarce, MntS helps manganese to activate a variety of enzymes. Its overproduction under manganese-rich conditions caused manganese to accumulate to very high levels inside the cell; simultaneously, iron levels dropped precipitously, apparently because manganese-bound Fur blocked the production of iron importers. Under these conditions, heme synthesis stopped, ultimately depleting cytochrome oxidase activity and causing the failure of aerobic metabolism. Protoporphyrin IX accumulated, indicating that the combination of excess manganese and iron deficiency had stalled ferrochelatase. The same chain of events occurred when mutants lacking MntP, the manganese exporter, were exposed to manganese. Genetic analysis suggested the possibility that MntS exerts this effect by inhibiting MntP. We discuss a model wherein during transitions between low- and high-manganese environments *E. coli* uses MntP to compensate for MntH overactivity, and MntS to compensate for MntP overactivity.

Author Summary

The model organism *Escherichia coli* can employ manganese to activate a variety of enzymes, but it does so only when iron is unavailable or the cell is assaulted by oxidants. Under those stress conditions, *E. coli* activates the synthesis of its MntH manganese importer. However, manganese can also be toxic, and over-accumulation of the metal must

be avoided. We investigated additional mechanisms by which the cell ensures that its intracellular manganese pool is sufficient but not excessive. MntS was identified as a small protein that is synthesized when manganese levels decline; it helps to enlarge the manganese pool. In contrast, MntP is a manganese exporter that is synthesized when intracellular manganese levels rise; it keeps manganese levels from becoming too high. In manganese-rich medium either the forced expression of MntS or the absence of MntP led to manganese toxicity, suggesting the possibility that MntS operates as an inhibitor of MntP. This toxicity occurs because excess manganese inhibits the synthesis of heme, a cofactor that is essential for aerobic growth. Thus *E. coli* controls manganese levels by balancing the actions of MntH, MntP, and MntS against one another. This arrangement allows cells to adapt to shifts in manganese availability and demand.

Introduction

Unstressed *Escherichia coli* appears not to use manganese as an enzyme cofactor. In this aspect the bacterium probably resembles ancient microbes, which evolved in an anoxic world whose metabolism was configured around the catalytic capabilities of iron. However, after the advent of photosystem II, molecular oxygen gradually accumulated in the atmosphere and caused the oxidation and precipitation of most environmental iron [1]. As a result, this metal is episodically unavailable in many contemporary habitats. Because bacteria inherited the ancestral metabolic machinery, they were forced to evolve strategies to cope with iron scarcity. For example, many bacteria synthesize siderophores to solubilize and import ferric iron [2]; during times of high iron availability they store any excess iron in ferritins to hedge against future scarcity [2]; and when iron is limited, they initiate an iron-sparing response, regulated by the small RNA (sRNA) RyhB, to prioritize iron use by shutting down the synthesis of iron-requiring enzymes that are abundant but not essential [3].

Enteric bacteria also engage an additional tactic: they compensate for iron deficiency by importing manganese to be used in its place. During periods of iron starvation the iron-sensing Fur repressor is deactivated, causing induction of the MntH manganese-uptake system [4,5]. At the same time *E. coli* also replaces two key iron-dependent redox enzymes—superoxide dismutase (FeSOD) and ribonucleotide reductase (NrdAB)—with their manganese-dependent isozymes (MnSOD and NrdEF), which poise manganese at the correct potential for catalysis [6,7,8]. Bacteria also employ a lone ferrous iron atom as a cofactor in a wide range of non-redox enzymes. These enzymes can be directly activated by manganese almost as well as by iron, and it appears that the induced import of manganese will allow these enzymes to retain function in iron-poor cells [9,10].

The MntH importer is also induced as an essential part of the OxyR response during periods of hydrogen peroxide (H_2O_2) stress [5,11,12]. The rationale is that H_2O_2 readily oxidizes the exposed ferrous cofactors of the same non-redox iron enzymes, leading to dissociation of ferric iron and inactivity [9,10]. Replacement of the iron with a manganese cation, which H_2O_2 cannot oxidize, appears to restore activity and sustain the function of the pathways to which these enzymes belong [9,10]. These observations suggest that *E. coli* relies upon manganese primarily when iron is scarce or H_2O_2 is present, and thus far growth defects have been documented for *mntH* null mutants only under those conditions [11,13].

However, the similarities between iron and manganese may also create problems for cells. Investigators have long recognized that high levels of extracellular manganese can inhibit bacterial growth [14,15]. The mechanism is not clear, but it seems likely that manganese might

cause problems by outcompeting iron for the metal-binding sites of proteins that cannot function with manganese. While in principle manganese might bind the mono- or bi-nuclear sites of redox proteins such the iron-dependent ribonucleotide reductase, the presence of complementary manganese isozymes would appear to forestall any metabolic disruption. However, other plausible targets include the iron-binding sites of ferrochelatase, which subsequently inserts ferrous iron into porphyrins in the final step of heme synthesis, and of the Isc iron-sulfur-cluster assembly machinery. Interference with either of these processes would ultimately diminish the activities of all the enzymes that utilize heme or iron-sulfur-cluster cofactors.

In this light it is not surprising that bacteria employ multiple devices to enforce upper limits upon manganese content. Manganese overloading is a potential threat when cells expressing the MntH manganese importer enter manganese-rich habitats, or when manganese enters the cell through less-specific divalent cation importers. MntR is a transcriptional factor that binds two Mn atoms in manganese-replete cells, and in the MntR:Mn_2 form it represses *mntH* transcription, thereby slowing synthesis of the importer [5,16,17]. Simultaneously MntR:Mn_2 induces synthesis of manganese efflux pumps. The identification of such pumps in *E. coli* (MntP), *Streptococcus pneumoniae* (MntE), and *Neisseria spp*. (MntX) demonstrated that bacteria strive to remove excess manganese before levels become toxic [18,19,20].

Recent transcriptomic evidence revealed the presence of a third member of the MntR regulon, MntS [20]. The *mntS* gene is expressed as an RNA that is predicted to have complex secondary structure; this RNA is termed RybA. Within the RNA lies a short open reading frame that encodes a small protein known as MntS. This protein is conserved and expressed. Transcription of *mntS* is repressed by MntR:Mn_2, suggesting that MntS plays a role in manganese homeostasis only when manganese is scarce. The nature of that role has not been clear. Mutants that lack MntS are defective at manganese-mediated repression of *mntH* transcription, as if the action of MntS can help MntR to acquire manganese. Conversely, strains that overexpress MntS are hypersensitive to exogenous manganese. These data suggest that when manganese is scarce, MntS may help make it available to potential manganese-binding proteins. One possibility is that the MntS protein, which is predicted to comprise 42 amino acids and seems too small to be an enzyme, might be a manganese chaperone that helps metallate enzymes; alternatively it might affect manganese content by perturbing the manganese influx or efflux systems.

In this study we explored the physiological role of MntS. We found that MntS synthesis elevates the total intracellular levels of manganese. This facilitates manganese binding to authentic client proteins but also exacerbates the ability of excess manganese to poison iron-specific cell functions, such as heme synthesis. The *mntS* overproduction phenotype matches that of an *mntP* deletion, suggesting the possibility that MntS may act as an inhibitor of that export system.

Results

MntS confers resistance to hydrogen peroxide during manganese limitation

The fact that MntS is expressed only when manganese levels are low suggested that it might help activate metalloenzymes during manganese limitation [20]. Manganese confers activity to non-redox mononuclear enzymes during H_2O_2 stress [9,10]. The role of MntS in this process was therefore examined.

To test a possible contribution of MntS to the activity of non-redox mononuclear enzymes, we examined cell growth during oxidative stress. *E. coli* Hpx^- mutants (*katG katE ahpCF*) cannot degrade H_2O_2 [21], and their growth in the presence of H_2O_2 requires the import of manganese [11]. Under this condition manganese uptake ensues when the OxyR regulator senses

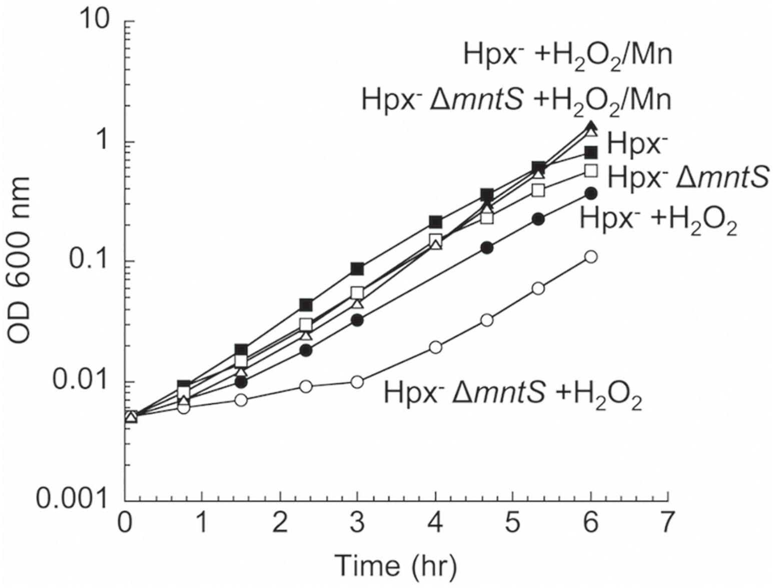

Fig 1. MntS confers resistance to hydrogen peroxide upon manganese limitation. Cells that cannot scavenge H_2O_2 (Hpx$^-$) were pre-cultured in anoxic M9 glucose/casamino acids medium and then diluted at time zero into aerated medium of the same composition. Where indicated, 15 μM H_2O_2 and/or 5 μM $MnCl_2$ were included in the aerated medium. Strains were LC106 (Hpx$^-$) and JEM1177 (Hpx$^-$ Δ*mntS*). The data are representative of at least three independent experiments.

doi:10.1371/journal.pgen.1004977.g001

H_2O_2 and induces MntH up to 50-fold [5]. In our standard defined medium, the *mntS* mutants exhibited a protracted lag when moderate (15 μM) amounts of H_2O_2 were supplied (Fig. 1). The lag was suppressed when high levels of manganese were included in the medium. The phenotype was complemented by a plasmid expressing *mntS* (S1 Fig.). This result suggests that MntS facilitated the activation of mononuclear enzymes by manganese during the period before MntH promoted manganese accumulation to high levels.

Using these conditions of oxidative stress, we tested whether MntS acts exclusively as an ancillary protein to either the MntR transcription factor or the MntH importer. MntS affects *mntH* expression, likely through MntR [20], but we observed that MntS helps outgrowth even in an *mntR* null background (S2 Fig., panel A). Moreover, MntH (panel B) and MntS (panel C) each confer growth benefits in the absence of the other. Thus MntS exerts an action that does not strictly depend upon MntR or MntH.

MntS facilitates manganese delivery to manganese-dependent enzymes during manganese limitation

The above data suggested that MntS somehow promotes manganese insertion into non-redox enzymes, but we were unable to test this idea directly because manganese dissociates from these enzymes during extract preparation. However, manganese is also an essential cofactor for the MnSOD superoxide dismutase [7,8,22]. Therefore, we examined the impact of MntS upon the metallation status of the mononuclear redox enzyme MnSOD under unstressed growth conditions. This enzyme closes around the bound manganese atom and does not allow it to dissociate in vitro [23]. In previous studies we demonstrated that this enzyme is only partially populated with manganese in our standard minimal medium [11], because the MntH manganese importer is minimally synthesized when iron is available [24]. Cell extracts were prepared, the MnSOD activity was assayed, and then the activity was assayed a second time after

reversible denaturation and reconstitution in the presence of manganese. The latter procedure fully activates the enzyme, and comparison of the pre- and post-reconstitution activities allowed us to appraise what fraction of the enzyme was initially active. In wild-type cells about 30% of the enzyme was active prior to reconstitution, while *mntS* mutants exhibited only 15% activity (Fig. 2A). Conversely, overexpression of the MntS protein from a plasmid caused full (~ 100%) MnSOD activation. These data confirmed that MntS helps make manganese available to enzymes.

Notably, both the Hpx⁻ and the Hpx⁻ *mntS* mutants exhibited full MnSOD activation (Fig. 2A). We suspected that this reflected the fact that H_2O_2-stressed cells contain high levels of manganese due to their robust MntH induction by OxyR. To see whether MntS is needed for MnSOD activation only at low intracellular levels of manganese, we examined its effect in *mntH* mutants that were supplemented with varying amounts of manganese. When MntH is absent, manganese may enter the cell less efficiently through other, non-specific divalent metal import systems [25,26]. We found that whereas supplementation with 0.4 μM manganese enabled full MnSOD activation in MntS-proficient strains, about 5-fold more manganese was needed in *mntS* mutants (Fig. 2B). These data demonstrate that MntS somehow assists in the metallation of MnSOD when intracellular manganese levels are low but is dispensable when levels are high.

Finally, we examined whether MntS helps to activate another manganese-dependent redox enzyme, NrdEF ribonucleotide reductase (7,8). NrdEF performance can be monitored when *nrdAB* null mutants are shifted from anoxic medium to aerated medium [8]. In this circumstance the oxygen-sensitive anaerobic NrdDG ribonucleotide reductase stops working, leaving NrdEF as the only ribonucleotide reductase that can function. Because iron competes with manganese for binding, NrdEF is activated only in iron-deficient cells, and so these studies were performed in strains lacking the Feo-, ferric-citrate-, ZupT-, and siderophore-dependent

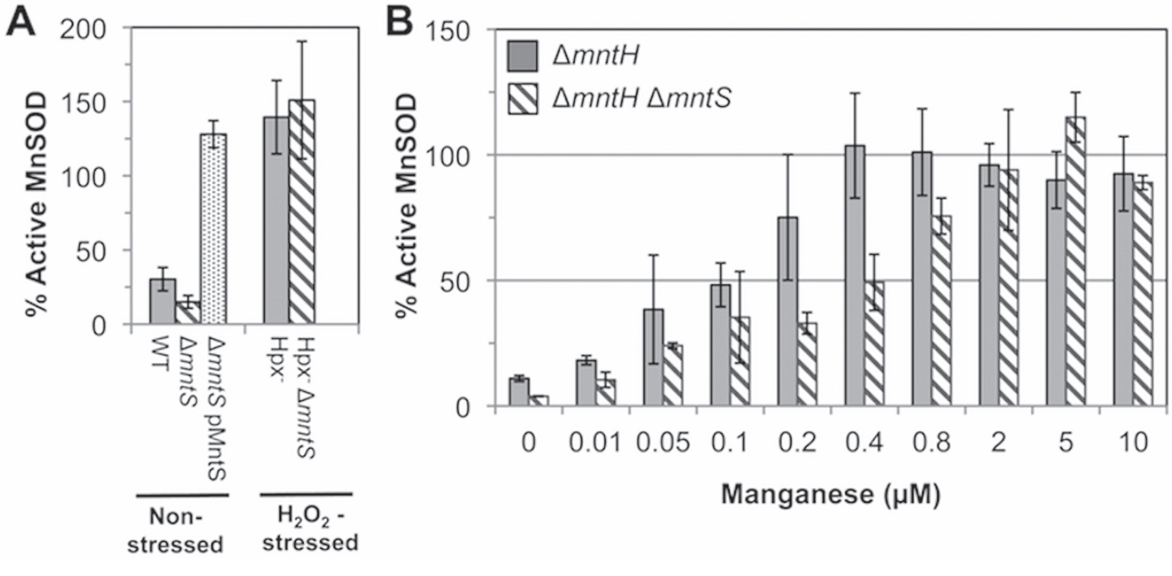

Fig 2. MntS facilitates the activation of manganese-dependent superoxide dismutase. The fraction of MnSOD protein that is catalytically active was measured in cell extracts prepared from cultures grown aerobically in M9 glucose/casamino acids media containing the indicated concentrations of $MnCl_2$. All strains contain the *sodB*-null allele. Data represent the mean of three independent cultures. A. MntS assists manganese insertion into MnSOD in manganese-poor cells. Strains were JEM1233 (WT), JEM1234 (Δ*mntS*), JEM1311 (Δ*mntS* with pMntS2, *mntS* under the control of its native promoter), JEM1208 (Hpx⁻), and JEM1212 (Hpx⁻ Δ*mntS*). The Δ*mntS* allele had minimal impact upon the amount of total MnSOD protein, as indicated by the activity after reactivation. B. MntS helps activate MnSOD when manganese concentrations are low. Strains were JEM1235 (Δ*mntH*) and JEM1237 (Δ*mntH* Δ*mntS*).

doi:10.1371/journal.pgen.1004977.g002

iron-import systems (Δ*tonB* Δ*feoABC* Δ*zupT*). Upon aeration this strain exhibits a protracted lag, during which iron is progressively depleted and MntH and NrdEF are induced, followed by outgrowth that requires the manganese-activated NrdEF [8]. Invariably this lag was slightly longer for *mntS*-deficient cells (Fig. 3A). More strikingly, the lag was greatly reduced when MntS was modestly overproduced and manganese was supplemented (Fig. 3B), suggesting that NrdEF was activated more rapidly. Experiments using *lacZ* fusions showed that *mntS* had no effect on the transcription or translation of the *nrdHIEF* operon (< 10% difference). We infer that during this transition period MntS enabled the activation of NrdEF. In toto our data indicate that when manganese influx is limited, MntS facilitates manganese binding to a variety of enzymes by an unspecified mechanism.

Overproduced MntS disrupts intracellular manganese and iron pools when cells are grown in manganese-rich medium

The *mntS* gene can be repressed by MntR:Mn_2. Previous work suggested that this control is important for cell fitness in manganese-rich environments, as wild-type *E. coli* cells overexpressing MntS from a heterologous promoter were observed to be sensitive to manganese on plates [20]. We confirmed that the phenotype also occurs in aerobic liquid medium and observed that growth characteristically failed after several generations (Fig. 4). Since the MntS overproduction phenotype depended upon high external concentrations of manganese, we examined the effect of high MntS levels upon the intracellular metal pools.

Wild-type cells grown in LB medium typically contained only about 5 μM total manganese (Fig. 5A). SOD activity measurements allow us to deduce that the majority of this manganese was incorporated into MnSOD (Materials & Methods). The intracellular manganese concentration rose to about 15 μM when MntS was overproduced. When manganese was supplemented in the medium (0.5 mM), manganese levels rose to ~ 35 μM in the wild-type strain and to ~ 140 μM upon MntS overproduction. The increase in intracellular manganese in the MntS-overproducing cells was also observed by whole-cell EPR; the six-peak spectrum represents manganese in its divalent state (S3 Fig.). This raised the possibility that toxicity arose from an excessively large pool of intracellular manganese. Further, the impact of MntS upon cellular manganese content favored a model that MntS acts by influencing either manganese import or export.

Under the same conditions, ICP-MS data revealed a 4-fold reduction in total intracellular iron (Fig. 5B). Most intracellular iron is incorporated into proteins, and so whole-cell EPR analysis was performed to specifically measure the pool of loosely bound, or free, intracellular iron. This is the iron pool that is expected to be available for the metallation of newly synthesized proteins. Manganese treatment lowered the amount of loosely bound iron in wild-type cells from ~100 to ~40 μM. However, the effect was much more severe when MntS was overproduced, as this iron pool fell from ~90 to ~2 μM (Fig. 5C).

We sought the reason for this collapse of the iron pool. Iron acquisition by *E. coli* is regulated by the transcription factor Fur; in its iron-metallated form, Fur:Fe inhibits synthesis of iron-import systems. Transcriptional fusions demonstrated that the combination of MntS overproduction and manganese supplementation essentially eliminated the expression of the two Fur-controlled genes that we tested, *iucC* and *fhuA* (Fig. 6). Deletion of Fur restored full expression, eradicating the effects of both manganese and MntS. The *fur* mutation also restored the intracellular iron pools (Fig. 5, B and C) and obviated the growth defect (Fig. 4). This phenotypic suppression resulted from restored iron import rather than from induction of the RyhB-mediated iron-sparing response, since Δ*ryhB* strains exhibited the same MntS/Mn toxicity and the same relief by *fur* deletion (S4 Fig.).

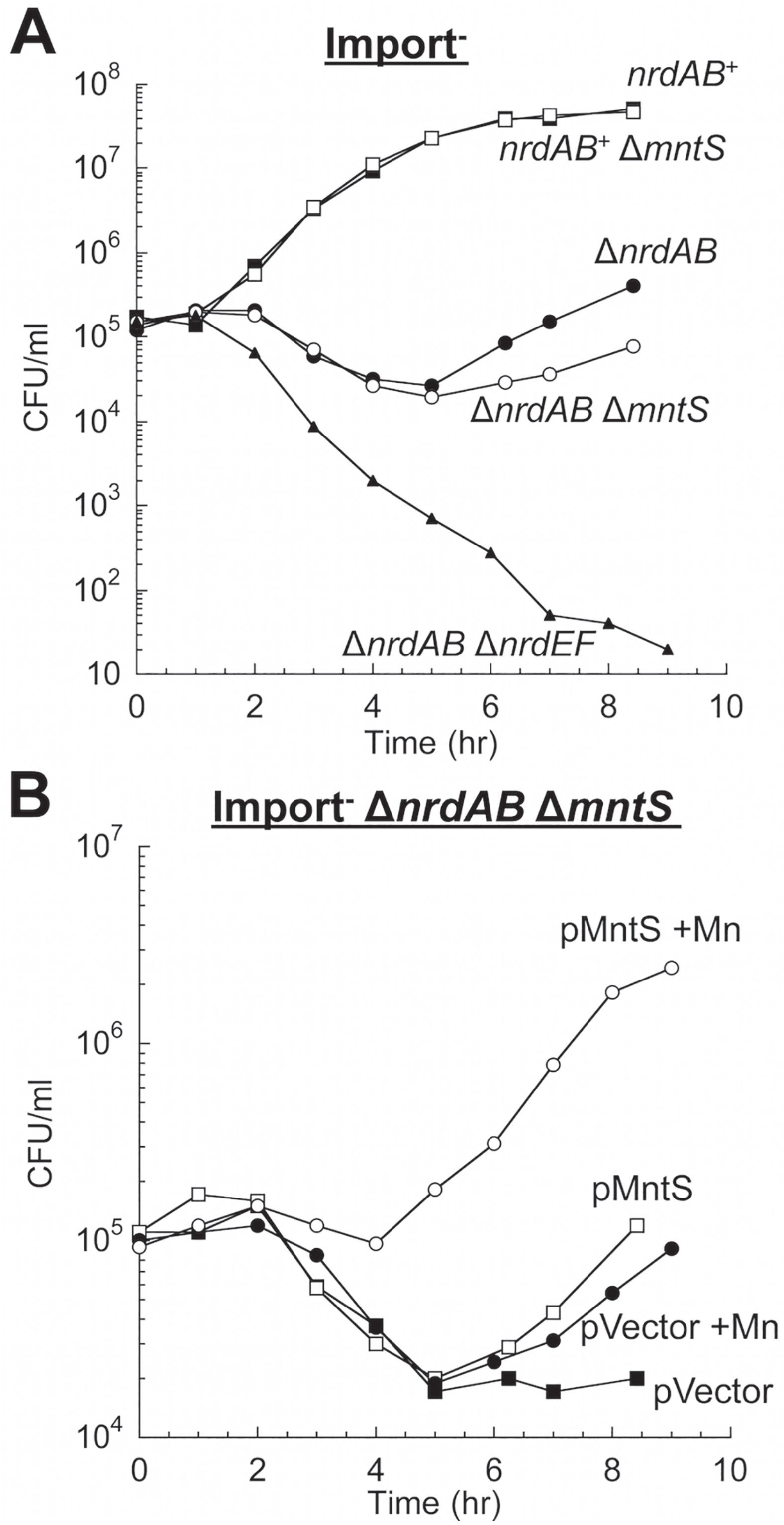

Fig 3. MntS helps activate the manganese-dependent RNR, NrdEF. Cells pre-cultured in anaerobic MOPS glucose/casamino acids medium were diluted into aerobic medium with or without 50 μM $MnCl_2$ at time zero; viability was monitored by anaerobic plating. "Import-minus" strains contain Δ*tonB* Δ*feoABC* Δ*zupT* null alleles and therefore have reduced iron import. The data are representative of at least three independent experiments. A. Strains were JEM609 (Import$^-$), JEM1181 (Import$^-$ Δ*mntS*), JEM1136 (Import$^-$

Δ*nrdAB*), JEM1836 (Import⁻ Δ*nrdAB* Δ*mntS*), and JEM722 (Import⁻ Δ*nrdAB* Δ*nrdHIEF*). B. Viability of Import⁻ Δ*nrdAB* Δ*mntS* (JEM1183) harboring empty vector (pACYC184) or pMntS2 (pLW131, *mntS* under the control of its native promoter).

doi:10.1371/journal.pgen.1004977.g003

Iron-bound Fur could not have mediated the repressive action of Fur in manganese-replete MntS-overproducing cells, since iron was vanishingly scarce. Instead, it is likely that Fur acted with Mn^{2+} as a cofactor. Manganese can substitute for iron in this protein in vitro and in vivo, and the Mn^{2+}-bound form of the protein is a capable repressor [4,5,27]. We anticipated that the consequent imbalance of high manganese and low iron was the likely source of toxicity.

Heme synthesis is inhibited by MntS overproduction

In *E. coli* iron activates mono- and di-nuclear iron enzymes, iron-sulfur proteins, and heme proteins. The combination of manganese overload and iron deficiency is unlikely to disrupt the pathways of the first group of enzymes, since the two redox-active iron enzymes—SOD and ribonucleotide reductase—can be replaced by manganese-using isozymes. Similarly, non-redox mononuclear iron enzymes would presumably not be inactivated by the iron depletion since they can typically be activated by manganese. However, manganese has never been observed to function in iron-sulfur or heme cofactors, and so we wondered whether excess manganese coupled with iron deficiency would compromise the synthesis of one or the other iron-dependent cofactor.

Iron-sulfur clusters are assembled upon the IscU scaffold protein and then transferred to client enzymes, including some that are essential for growth [28]. It seemed possible that manganese might competitively inhibit the entry of iron into this process or might simply block the process through the depletion of iron pools. We assayed the activity of NADH dehydrogenase

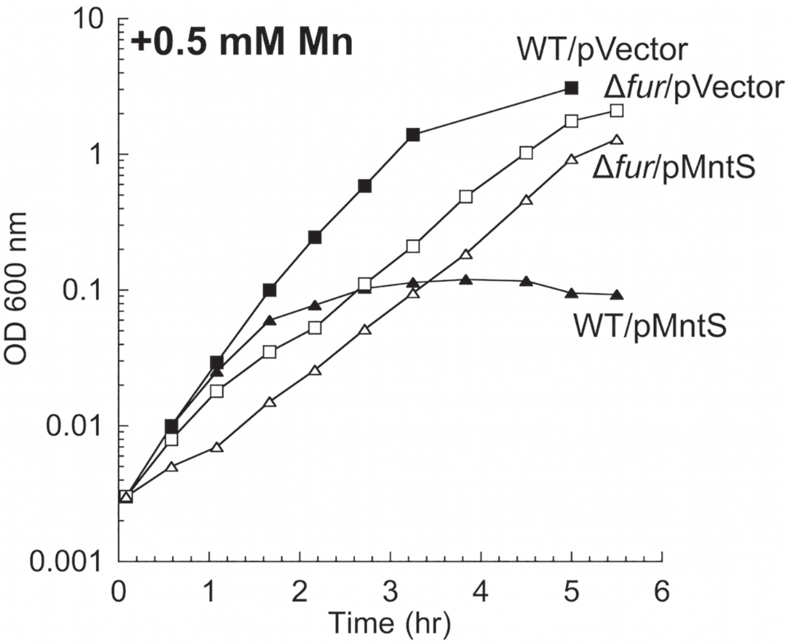

Fig 4. MntS-overproducing cells are manganese sensitive. Cells were pre-cultured in aerobic LB medium and then diluted at time zero into fresh LB/arabinose medium containing 0.5 mM $MnCl_2$. Strains were MG1655 (WT) and JEM913 (Δ*fur*) harboring empty vector (pBAD24) or pMntS (pLW112, *mntS* driven by the *araBAD* promoter). The data are representative of at least three independent experiments.

doi:10.1371/journal.pgen.1004977.g004

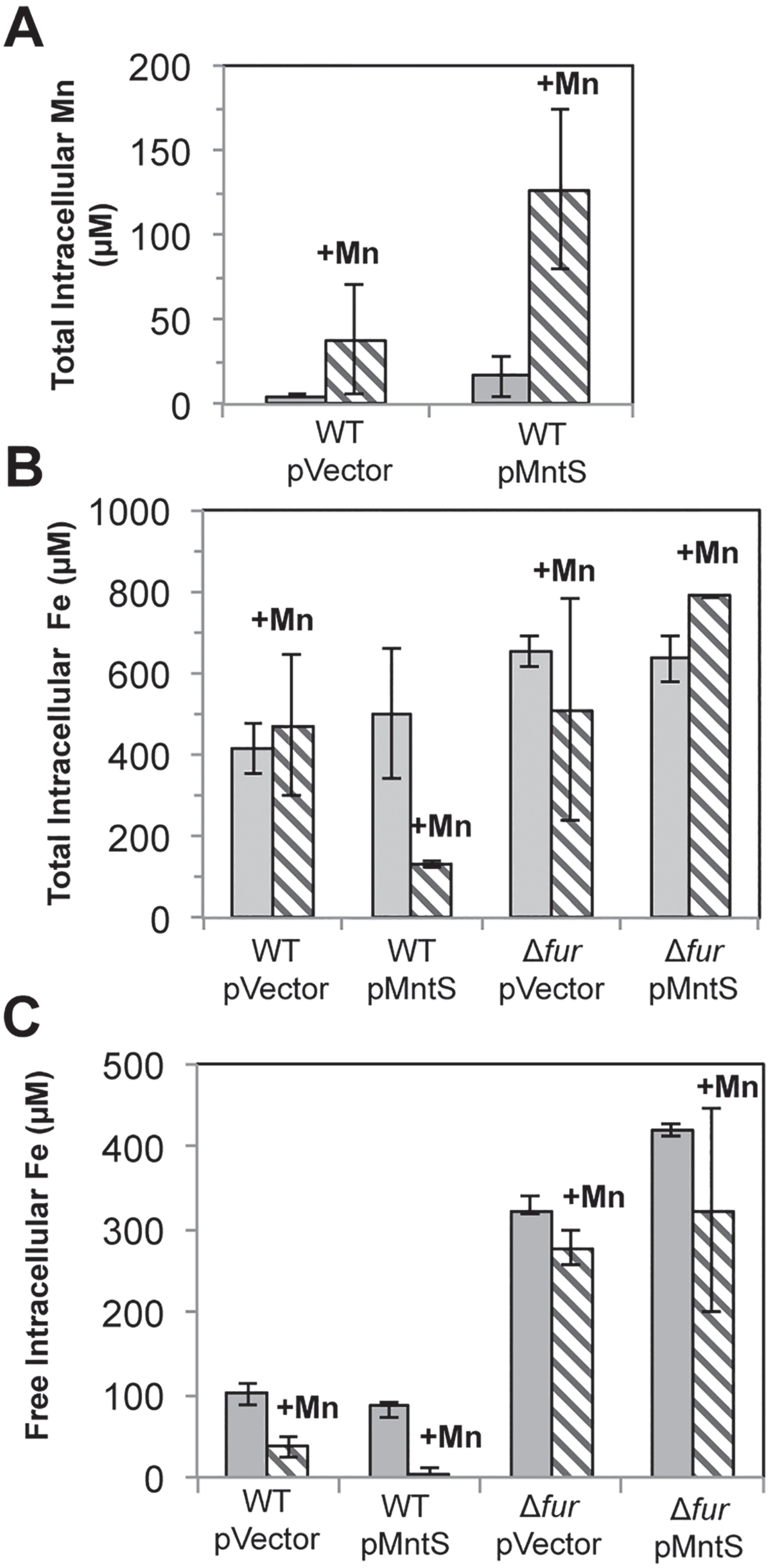

Fig 5. Overproduced MntS disrupts metal pools in manganese-rich medium. Cells pre-cultured in aerobic LB medium were diluted into fresh LB/arabinose medium with or without 0.5 mM $MnCl_2$ and harvested after 2.5 hr of aerobic growth. Data represent the mean of three independent cultures. Strains were MG1655 (WT) and JEM913 (Δ*fur*) harboring empty vector (pBAD24) or pMntS (pLW112, *mntS* driven by the

araBAD promoter). A. Total intracellular manganese measured by ICP-MS. B. Total intracellular iron measured by ICP-MS. C. The concentration of unincorporated intracellular iron measured by EPR spectroscopy.

doi:10.1371/journal.pgen.1004977.g005

I, a respiratory enzyme that requires nine iron-sulfur clusters for function. This enzyme activity is sharply diminished in cells that have even partial defects in iron-sulfur assembly [29,30]. However, NdhI activity remained at normal levels during manganese intoxication (S5A Fig.). Further, there was no increase in the transcription of the *iscR* and *sufA* genes (S5C Fig.). These genes are strongly induced when iron-sulfur synthesis is hindered, due to conversion of the IscR[2Fe-2S] transcription factor to its apoprotein form [31]. Collectively, these data indicate that excess manganese did not disrupt iron-sulfur assembly, and so the growth defect did not result from the loss of an iron-sulfur enzyme.

Heme is a cofactor for relatively few enzymes in *E. coli*: catalases G and E, cytochrome o and bd oxidases, succinate dehydrogenase, and the nitrite and sulfite reductases (S3 Table). Assays revealed that catalase G activity was 3.5-fold lower in manganese-fed cells overexpressing MntS compared to those carrying the empty vector (Fig. 7A). This activity in the MntS producers was measured when growth had slowed but not yet stopped. The low activity did not result from diminished transcription of *katG* (S6 Fig.), suggesting that it might stem from an impaired ability to activate the protein.

We then tested cytochrome oxidase activity by measuring the NADH oxidase activity of inverted cell membrane vesicles. At the pre-stasis time point, membranes prepared from cells overproducing MntS in the presence of high manganese showed 3-fold lower NADH oxidation compared to cells expressing empty vector, indicating a deficiency of cytochrome oxidase activity (Fig. 7B). NADH oxidase activity also depends upon upstream non-heme enzymes, NADH dehydrogenase I or NADH dehydrogenase II, but their activities were not diminished (S5A, B Fig.). Assuming the synthesis of the oxidase proteins was not affected, these data suggested that MntS interfered with heme production.

We examined possible points of inhibition in the heme biosynthetic pathway (S7A Fig.). Deletion of *hemA*, which encodes the rate-limiting first enzyme of the pathway, produced a strain that required 5-aminolevulinate, the product of HemL, to grow in aerated medium (S7B Fig.).

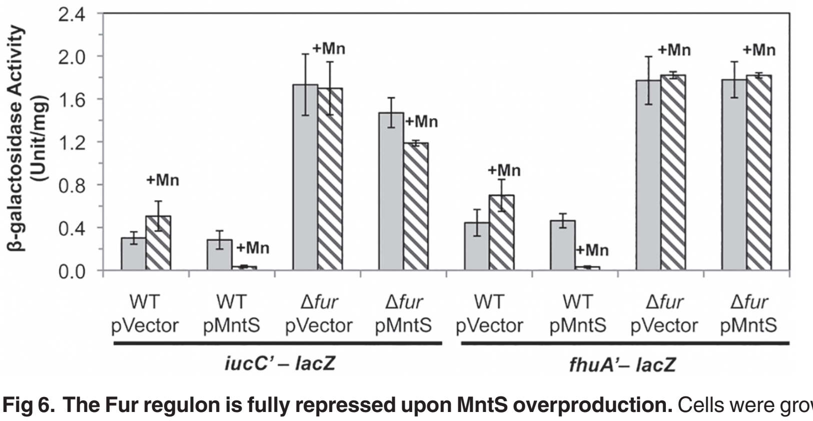

Fig 6. The Fur regulon is fully repressed upon MntS overproduction. Cells were grown in aerobic LB/arabinose medium with or without 0.5 mM $MnCl_2$ and aerated for 2.5 hr before harvesting. Data represent the mean of three independent cultures. Strains bearing *iucC'-lacZ* were JEM1369 (WT/pBAD24), JEM1370 (WT/pLW112), JEM1463 (Δ*fur* /pBAD24), and JEM1464 (Δ*fur*/pLW112). Strains bearing *fhuA'-lacZ* were JEM1395 (WT/pBAD24), JEM1396 (WT/pLW112), JEM1465 (Δ*fur* /pBAD24), and JEM1466 (Δ*fur*/pLW112). The pMntS (pLW112) plasmid encodes *mntS* driven by the *araBAD* promoter.

doi:10.1371/journal.pgen.1004977.g006

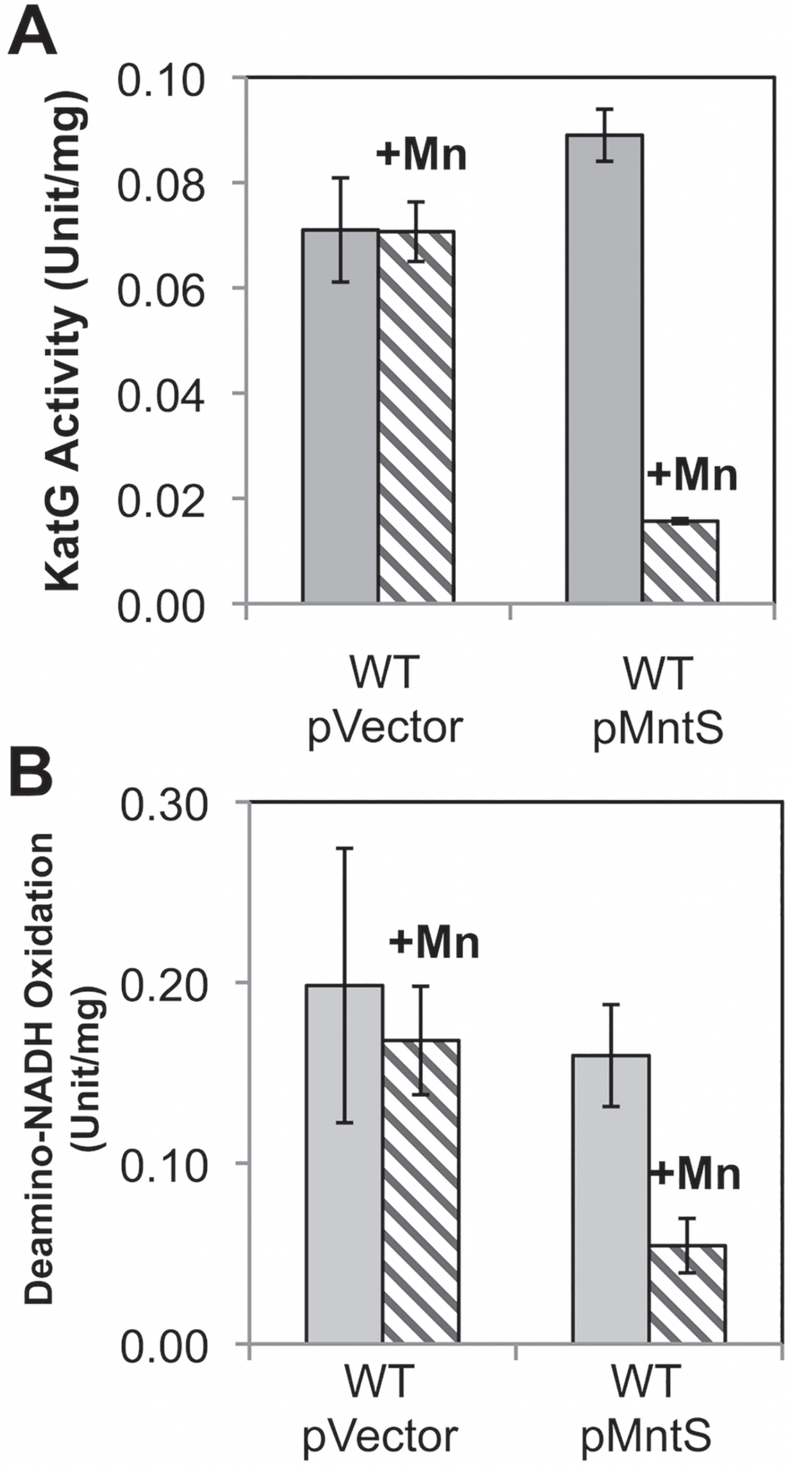

Fig 7. Heme-containing enzymes, catalase and cytochrome oxidase, fail to function properly during manganese toxicity. Cells were grown in anaerobic LB/arabinose medium with or without 0.5 mM $MnCl_2$ and aerated for 2.5 hr before harvesting. Data represent the mean of three independent cultures. A. KatG activity was determined from strains JEM1405 (WT/pBAD24) and JEM1406 (WT/pLW112). B. Deamino-NADH oxidation was determined from JEM1397 (WT/pBAD24) and JEM1398 (WT/pLW112). These strains lack the back-up iron-sulfur *suf* operon in order to demonstrate that the failure of heme synthesis was not accompanied by defects in iron-sulfur assembly by the Isc system (S4A Fig.).

doi:10.1371/journal.pgen.1004977.g007

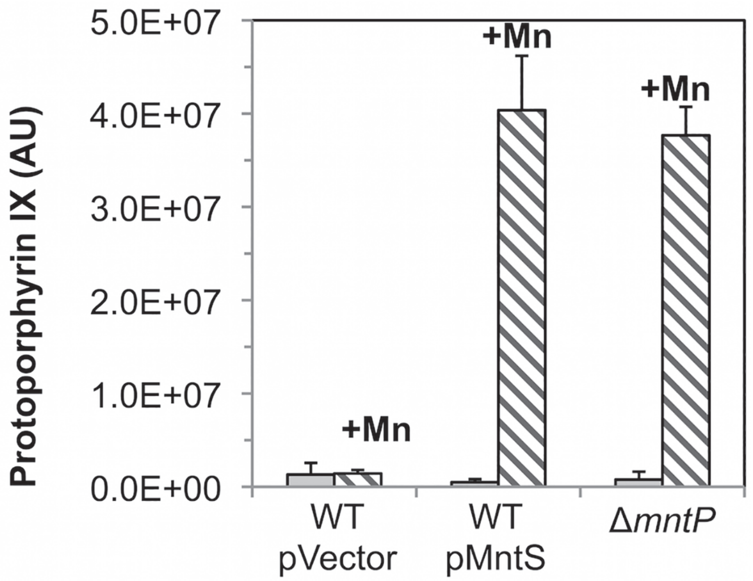

Fig 8. Ferrochelatase is inhibited by excess manganese. Cultures were grown in LB medium; where indicated, medium was supplemented for the final 2.5 hours with 0.5 mM $MnCl_2$. The intracellular levels of protoporphyrin IX were quantified by LC-MS-MS. Data represent the mean of three independent cultures. Strains were MG1655/pBAD24 (WT), JEM1406 (WT/pMntS), and MS025 (Δ*mntP*).

doi:10.1371/journal.pgen.1004977.g008

The strain grew without this supplement in anoxic medium, when respiration is dispensable. Notably, 5-aminolevulinate supplementation did not suppress the manganese sensitivity of *hemA* cells overexpressing MntS (S7B Fig.); thus the manganese-induced block was apparently downstream of HemL. Indeed, these cells accumulated 10-fold more metal-free porphyrins than did cells containing the empty vector (S8 Fig.).

A similar effect occurred when cells were treated with dipyridyl, a cell-permeable iron chelator that is likely to inhibit iron insertion into protoporphyrin IX by ferrochelatase. This is the final step in heme synthesis. To evaluate whether ferrochelatase is the precise target of manganese toxicity, LC-MS-MS analysis was performed upon extracts of otherwise wild-type cells that overexpressed MntS. The level of intracellular protoporphyrin IX was elevated ~ 80-fold under conditions of manganese poisoning (Fig. 8). In contrast, levels were normal in cells that did not overproduce MntS or that were not supplemented with manganese. Thus ferrochelatase (HemH) fails when the iron/manganese balance is perturbed.

Features of the growth defect can be explained by the fact that excess manganese specifically disrupts heme synthesis and cytochrome oxidase activity. Growth inhibition occurs gradually rather than immediately (Fig. 4), no matter what dose of manganese is added. This pattern is characteristic of interruptions in cofactor synthesis; withdrawal of vitamins from thiamine, biotin, or lipoate auxotrophs, for instance, did not impair growth for up to five generations (S9A Fig.). In the present example, once heme synthesis is blocked, several generations are required to dilute the titers of extant enzyme to the point that growth fails. This behavior was reproduced by *hemA* mutants when 5-aminolevulinic acid was withdrawn (S9B Fig.). Cytochrome oxidases are the only heme proteins that are critical for growth in aerobic LB medium (Mancini and Imlay, submitted; [32]). Indeed, MntS overproduction and manganese supplementation did not interfere with the growth of cells at all under anoxic conditions, a situation in which NADH oxidation is achieved by fermentative enzymes rather than by respiration (S10 Fig.).

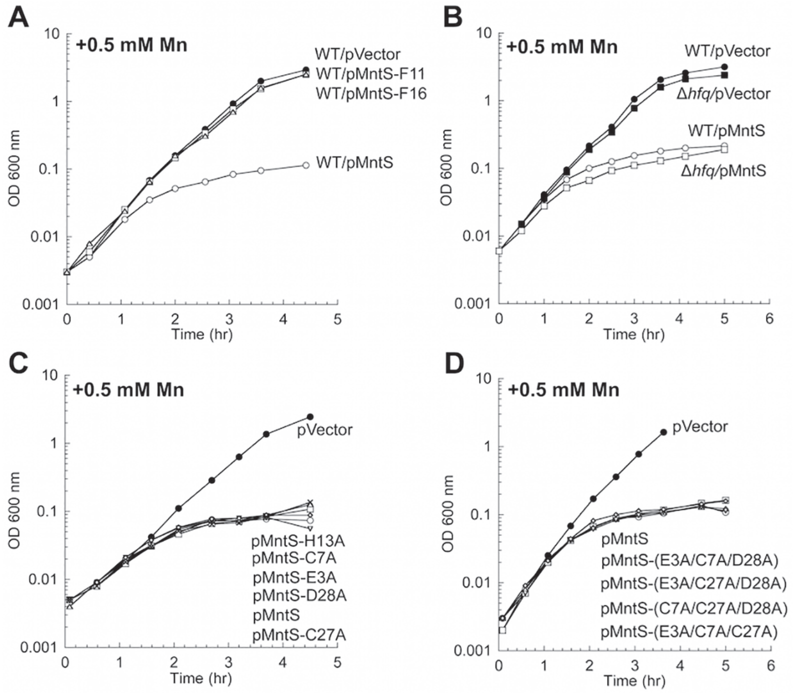

Fig 9. MntS confers manganese sensitivity by acting as a protein but not as a metal chaperone. Cells were cultured in aerobic LB/arabinose supplemented with 0.5 mM $MnCl_2$ 2.5 hr before harvesting. Data represent the mean of three independent cultures. A. Growth of WT (MG1655) cells expressing plasmids pVector (pBAD24), pMntS (pLW112, *mntS* driven by the *araBAD* promoter), pMntS-F11 (pJEM67, *mntS* containing a frameshift at F11), or pMntS-F16 (pJEM68, *mntS* containing a framshift at F16). ver time of Data represent the mean of three independent cultures. re JEM (s were harvested 2.5 hr mino acids meB. Growth of WT (MG1655) and Δ*hfq* (JEM1407) mutants. C. Growth of WT (MG1655) expressing plasmids with mutations in potential metal-binding residues: pMntS-E3A (pMS017), pMntS-C7A (pMS018), pMntS-C27A (pMS020), pMntS-D28A (pMS021), and pMntS-H13A (pLW125). D. Growth of WT (MG1655) expressing plasmids with combined mutations in potential metal-binding residues: pMntS-(E3A/C7A/D28A) (pLW133), pMntS-(E3A/C27A/D28A) (pLW134), pMntS-(C7A/C27A/D28A) (pLW135), or pMntS-(E3A/C7A/C27A) (pLW136).

doi:10.1371/journal.pgen.1004977.g009

Overproduction phenotypes are due to the MntS protein

The *mntS* ORF lies within the gene encoding RybA, which a computational study originally identified as a possible sRNA [33]. Northern blots showed seven mRNA species ranging from 205 to ~400 nucleotides; these have a common 5' end and all include the short *mntS* ORF [20]. The ORF is recognizable only within closely related enterobacteria; in principle, homologs might exist in more-distant organisms but be unrecognizable due to drift within its very short coding region. Still, analysis of the *mntS* sequences of these enterobacteria [18] indicates strong conservation of the ORF, as silent substitutions greatly predominate over non-silent ones. Only two of fifteen single-base changes observed among homologs result in codon switches. Further, Shine-Dalgarno sequences and stop codons are maintained at appropriate positions. Lastly, a tagged derivative of the protein expressed from the chromosome was readily detected upon manganese limitation. Nevertheless, one study has suggested that the transcript might function as a regulatory sRNA to control gene expression during oxidative stress [34].

It was not clear whether the *mntS* gene effected manganese poisoning by acting as a regulatory sRNA, by encoding a protein, or both. To test this idea experimentally, we constructed two separate frame-shift mutants of *mntS*, at Phe11 and Phe16. Cells expressing pMntS-F11 and pMntS-F16 alleles were no longer sensitive to manganese (Fig. 9A). The Fur regulon was

also significantly de-repressed (S11A Fig.), and catalase G activity was greatly increased (S11B Fig.). These data suggested that in this circumstance *mntS* was functioning as a protein rather than as a sRNA. Further, while sRNAs typically require the RNA chaperone Hfq in order to function [35], MntS imposed manganese toxicity equally in Δ*hfq* mutants and wild-type cells (Fig. 9B). Taken together, these data indicate that *mntS* most likely exerts manganese toxicity through its action as a small protein. This conclusion does not exclude the possibility that RybA acts as a sRNA in other contexts.

Lack of the MntP manganese exporter mimics MntS overproduction

In principle overproduced MntS could have elevated the level of intracellular manganese (Fig. 5A) by increasing its rate of influx, by delivering manganese to proteins that bind it, or by inhibiting export. The first possibility is unlikely, because MntS can impose an effect even in the absence of the primary manganese importer, MntH (S2B, C Fig.). The second possibility seemed unlikely because MntS affects the total amount of manganese in the cell. Nevertheless, we examined the idea that MntS might act as a manganese chaperone within the cell. We tested whether purified MntS would facilitate the metallation of MnSOD apoprotein in vitro, but we saw no effect (S12 Fig.). That result is not definitive, as it was possible that the purified protein was not functional. However, mutation of its Glu, Cys, Asp, and His residues—individually or as in combinations—did not eliminate the ability of MntS to confer manganese toxicity (Fig. 9C, D). Since these constitute the only plausible metal-binding residues on MntS, these results indicate that this activity of MntS does not require manganese binding.

The *mntP* gene is, alongside *mntH* and *mntS*, the third member of the MntR regulon [20]. Manganese-loaded MntR:Mn_2 induces *mntP* when manganese levels rise. MntP is a manganese-efflux pump, and *mntP* mutants were sensitive to manganese on solid media. This phenotype matches that of MntS overexpression, suggesting that MntS and MntP might act in the same pathway. The manganese sensitivity of Δ*mntP* mutants recurred in liquid medium and like that of the MntS overexpressors was relieved by deletion of *fur* (Fig. 10A). Further, manganese-supplemented Δ*mntP* mutants contained elevated levels of manganese and very low levels of loose iron (Fig. 10B, C). The *iucC* and *fhuA* genes were repressed by Fur (Fig. 10D), catalase G activity was reduced 5-fold (Fig. 10E), and protoporphyrin IX accumulated to levels similar to those of cells overproducing MntS (Figs 8 and 10F). Thus deletion of *mntP* fully phenocopies MntS overproduction.

All of these observations supported the notion that MntS might confer manganese sensitivity by inhibiting MntP function. We then overexpressed *mntS* in Δ*mntP* mutants, to examine whether MntS had any further effect that did not depend upon MntP. We observed no further change in the concentrations of intracellular manganese, iron, and porphyrins in Δ*mntP* mutants overexpressing MntS compared to those carrying the empty vector (S13 Fig.). Furthermore, the overproduction of MntS in Δ*mntP* mutants did not increase their manganese sensitivity, even when cells were grown with lower manganese concentrations (Fig. 11). Taken together, these data indicate that the *mntP*-null deletion and overexpression of MntS act in the same pathway to exert manganese toxicity. Thus, MntS may elevate intracellular manganese levels by inhibiting manganese export through MntP (Fig. 12). When the environmental level of manganese drops to lower levels, MntS inhibition of MntP could modestly increase the intracellular manganese pool and thereby enhance manganese entry into proteins. However, when external manganese is abundant, persistent inhibition of MntP would result in the excessive accumulation of manganese, which toxifies the cell by blocking iron import and preventing heme synthesis.

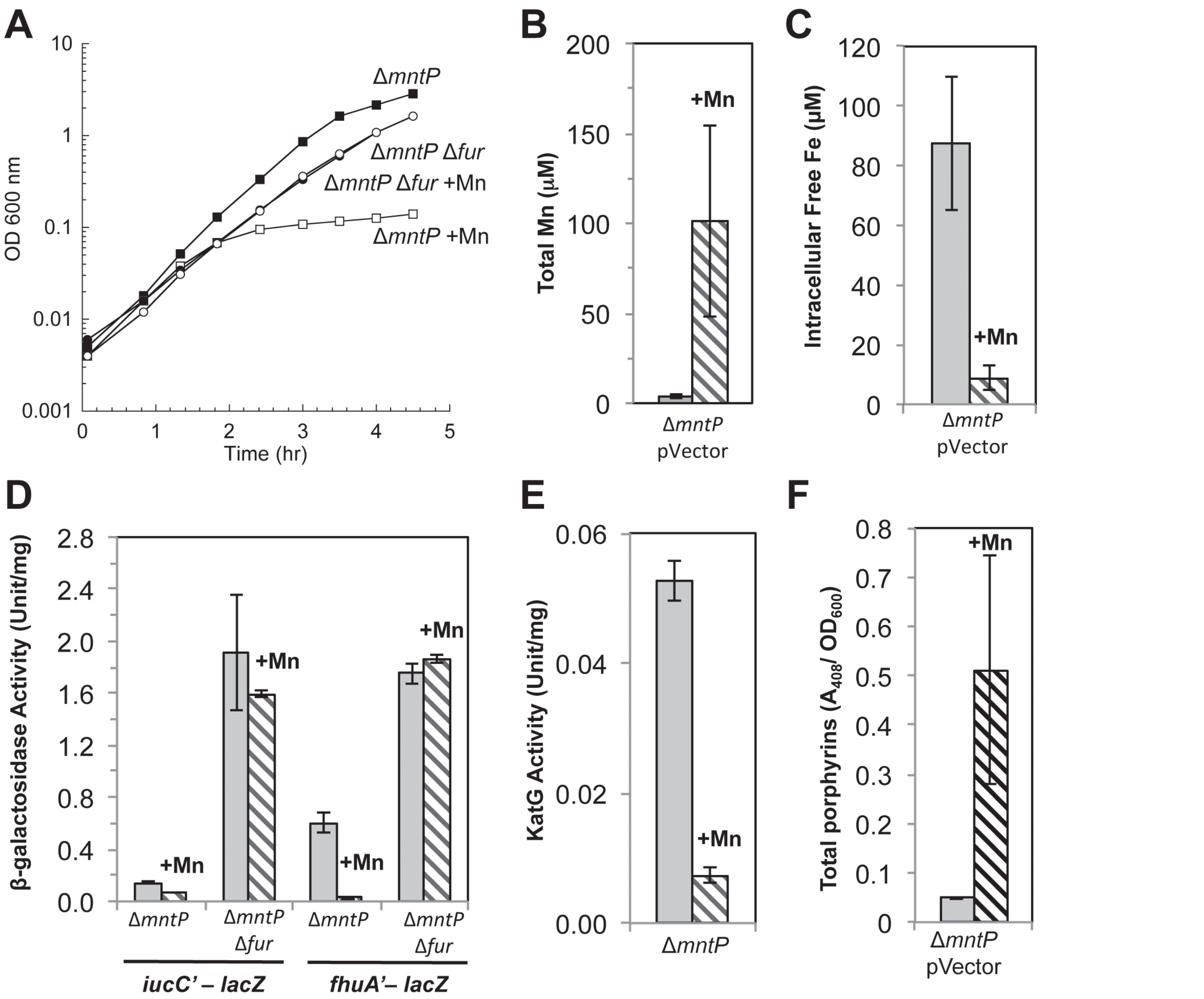

Fig 10. Lack of MntP, the manganese exporter, reproduces the phenotypes of MntS overproduction. Cells were grown in aerobic LB medium with or without 0.5 mM $MnCl_2$. Metal concentrations and enzymatic activities were measured after 2.5 hr of growth. Data represent the mean of three independent cultures. A. Growth of JEM1720 (Δ*mntP*) and JEM1722 (Δ*mntP Δfur*). B. Total intracellular manganese was determined by ICP-MS from JEM1726 (Δ*mntP*/pBAD24). C. Intracellular unincorporated iron was determined by EPR using JEM1726 (Δ*mntP*/pBAD24).D. Transcription levels of the Fur-regulated genes *iucC'-lacZ* or *fhuA'-lacA* from strains JEM1719 (Δ*mntP*) and JEM1724 (Δ*mntP Δfur*) or JEM1720 (Δ*mntP*) and JEM1722 (Δ*mntP Δfur*), respectively. E. Catalase G activity from MS025 (Δ*mntP*). F. Porphyrin accumulation in JEM1726 (Δ*mntP*/pBAD24).

doi:10.1371/journal.pgen.1004977.g010

Inhibition of MntP could occur directly through the binding of MntS to MntP, or indirectly through inhibition of MntP synthesis or another molecule required for MntP activity. Efforts to test physical binding between MntS and MntP were stymied by an inability to generate active antibodies against this very small hydrophobic protein. We did examine the impact of MntS upon MntP levels. The toxic impact of MntS overexpression was not mediated by any effect upon MntP synthesis, as an *mntP-lacZ* translational fusion was similarly induced 3- to 4-fold upon Mn supplementation both without and with MntS overexpression. Moreover, MntS

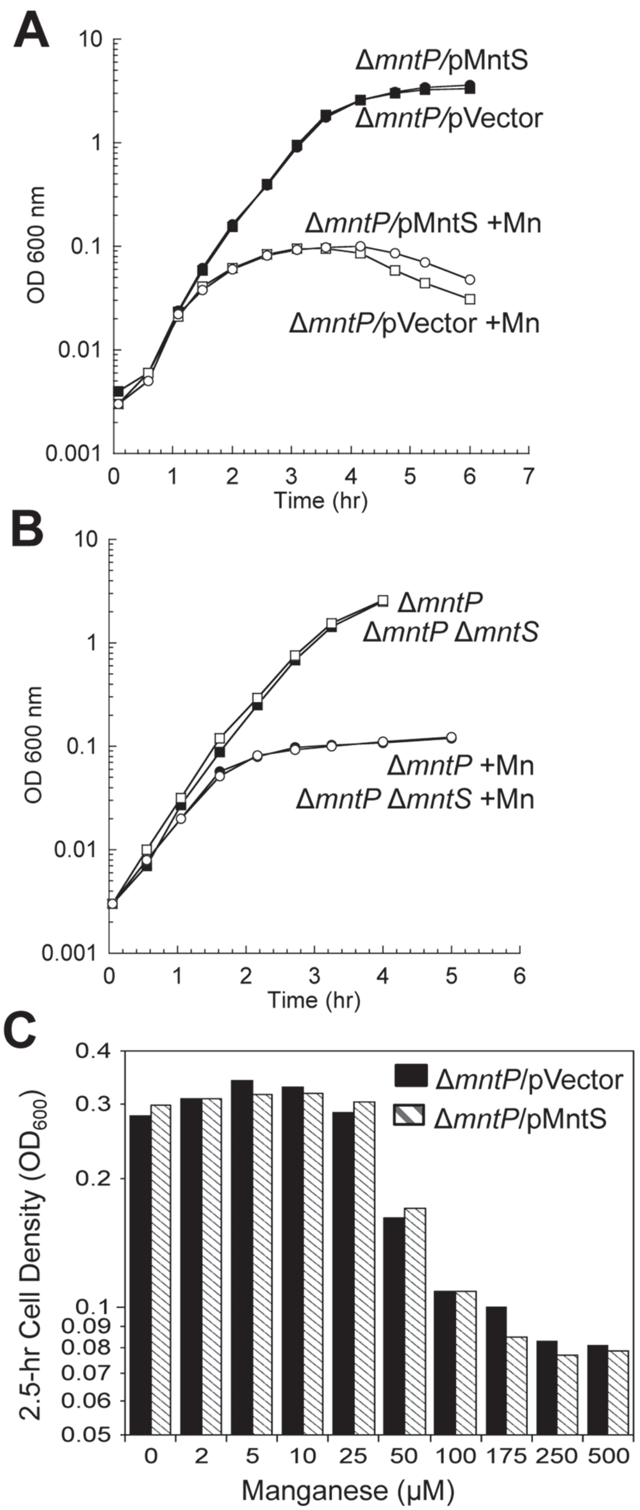

Fig 11. The *mntP*-null mutation is epistatic to overproduction of MntS with respect to manganese sensitivity. Cells were pre-cultured in aerobic LB medium and then diluted at time zero into fresh LB/arabinose medium with or without 0.5 mM $MnCl_2$. The data are representative of at least three independent experiments. A. Strains were JEM1726 (Δ*mntP*/pBAD24) and JEM1727 (Δ*mntP*/pLW112, *mntS* driven by *araBAD* promoter). B. Strains were MS025 (Δ*mntP*) and JEM1715 (Δ*mntP* Δ*mntS*). C. Growth density determined after growth with manganese for 2.5 hr. Strains were JEM1726 (Δ*mntP*/pBAD24) and JEM1727 (Δ*mntP*/pLW112, *mntS* driven by *araBAD* promoter).

doi:10.1371/journal.pgen.1004977.g011

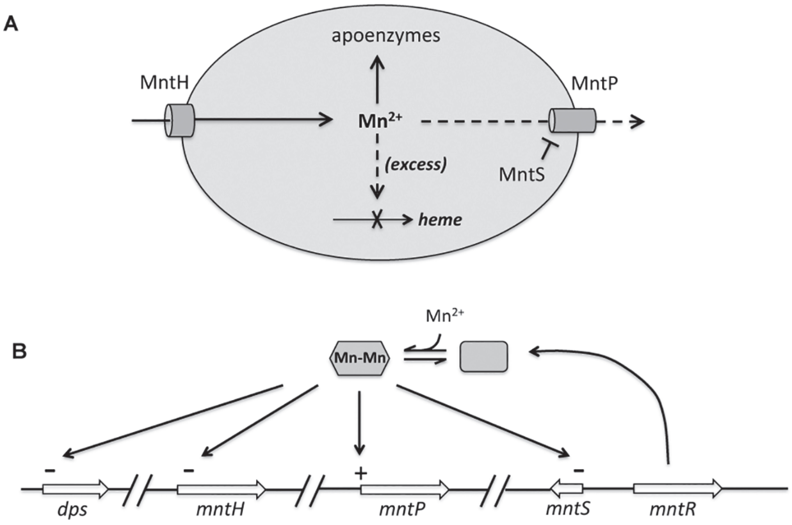

Fig 12. Proposed model of the MntR regulon. A. When expressed, MntH provides sufficient manganese for enzyme metallation. Excessive manganese (dashed lines) potentially inhibits heme synthesis at the ferrochelatase step, and so induction of MntP is needed to export manganese. When manganese levels fall, induction of MntS inhibits MntP and maintains manganese pools. B. Transcriptional effects of manganese-loaded MntR. The *mntH* gene is additionally repressed by iron-loaded Fur, and both it and *dps* are induced by H_2O_2-oxidized OxyR.

doi:10.1371/journal.pgen.1004977.g012

synthesis did not diminish the amount of total MntP protein in the cell (S14 Fig.), ruling out an impact upon MntP synthesis or stability transcription, translation, or protein stability. We hypothesize that MntS elevates cellular manganese levels by directly or indirectly inhibiting MntP exporter activity. However, in vitro physical studies likely will be needed to test this hypothesis.

Discussion

The recognition that manganese blocks heme synthesis helps to complete our understanding of how excessive amounts of various first-row transition metals can impair cell fitness. Excess iron is detrimental due to its participation in Fenton chemistry [36,37]. In contrast, many of the other metals are toxic primarily because they compete for one another's binding sites; thus the optimal intracellular level of one transition metal depends upon the ambient levels of others. Excess cytoplasmic copper can poison *E. coli* by displacing iron atoms from iron-sulfur clusters [38,39], while divalent zinc can disable mononuclear iron enzymes by binding to the ferrous site [40]. Cobalt disrupts iron-sulfur assembly by outcompeting iron for the Isc scaffold system [41], and nickel poisons *E. coli* by binding to zinc and iron sites in enzymes [42,43]. In contrast, manganese has a more complicated, intermediate role: when iron is scarce or vulnerable to Fenton chemistry, manganese is a useful substitute for iron in many metalloproteins, and so manganese import is induced. However, too much manganese is a problem, because it can outcompete iron for proteins that manganese cannot activate. This study of *E. coli* demonstrates the involvement of MntS in ensuring manganese sufficiency, the role of MntP in avoiding excess, and the consequences when this system is disrupted.

The mechanism of Mn toxicity

E. coli is not known to use manganese in standard defined lab media: the MntH manganese importer is repressed, little manganese accumulates in the cell, the few manganese-specific enzymes are inactive, and deletion of *mntH* is without apparent phenotypic consequence.

However, when iron is unavailable or H_2O_2 is present, mononuclear iron enzymes may lose activity, and manganese import becomes critical for cell fitness. This fact has been demonstrated by the phenotypes of *mntH* mutants [11,13]. Here, milder versions of the same defects were exhibited in *mntS* mutants through the poor performances of ribonucleotide reductase during iron starvation and of mononuclear enzymes during H_2O_2 stress.

At the other end of the spectrum is manganese overloading. One can infer that too much manganese is problematic from the prevalence of manganese-export systems among microbes. By examining the growth defects of *mntS* overexpressors and of *mntP* null mutants, we have learned that excessive intracellular manganese drives the pool of unincorporated iron down to a level that cannot sustain heme synthesis. We suggest that when iron levels are this low, manganese competitively inhibits the action of ferrochelatase. The accumulation of protoporphyrin IX supports this suggestion, and other workers have shown that purified mammalian ferrochelatase inserts Mn into protoporphyrin IX and then fails to release the product, thus stalling the enzyme [44,45]. Analogous experiments in our lab have been thwarted thus far by the aggregative tendency of the *E. coli* enzyme. In contrast, manganese is not known to form manganese-sulfur clusters analogous to iron-sulfur clusters, presumably because it binds sulfur much more poorly than does iron [46]; this may explain why heme synthesis is inhibited more readily than is cluster synthesis.

The drop in iron levels is mediated in part by the repression of iron-import systems, presumably by Fur:Mn complexes. But iron deficiency cannot be ascribed entirely to this action, since mutants that are devoid of the known Fur-repressible importers (*feo*, *fec*, *ent*) are still capable of synthesizing heme and grow well in the LB medium that we used. We think two other aspects of manganese poisoning exacerbate the import defect. First, repression of the Fur regulon by Fur:Mn also blocked expression of RyhB (S15 Fig.), the sRNA that shuts down the synthesis of expendable iron proteins in order to spare iron for necessary proteins [47]. Cells lacking RyhB cannot cope with iron deficiency; they exhibit very small loose-iron pools and grow poorly [48], similar to the behavior of the Mn-poisoned cells of this study. Further, it is possible that high extracellular manganese levels competitively inhibit secondary iron import by binding to non-specific divalent-cation importers. Additionally, intracellular manganese may stall the same transporters through product inhibition, forming complexes with their metal-binding sites when they are cytoplasmically exposed and thereby blocking their cycling back to periplasmic exposure.

In sum, the ability of manganese to occupy iron-binding sites has both productive and destructive consequences. In *E. coli* moderate levels of manganese rescue the activities of mononuclear iron enzymes when iron is scarce, but high levels inappropriately activate Fur and eliminate heme synthesis. It is interesting to consider how microbes that routinely employ both metals are able to balance their use. *Bradyrhizobium japonicum* is an obligate aerobe that cannot grow if either metal is lacking. O'Brian and co-workers have shown that manganese activates its sole cytoplasmic superoxide dismutase, and manganese also cofactors its pyruvate kinase [49]. Significantly, this bacterium controls iron import not through a Fur-based system, which clearly lacks specificity for iron, but through Irr, a positive transcription factor that measures heme pools instead [50]. Irr stability is enhanced by bound manganese [51]. This device offers two mechanisms that circumvent the inhibition of heme synthesis by manganese: iron import is stimulated both when manganese levels rise and/or when heme levels drop. Lactic acid bacteria comprise another instructive example. They are exceptional among bacteria in that they maintain very high intracellular manganese:iron ratios [52,53]—but their metabolic design allows them to tolerate this situation because they typically do not attempt to synthesize heme. They either do not employ heme at all or else acquire it from import [54].

Multiple steps to control manganese levels

MntP and MntS provide new layers of manganese control. We have known for some time that when iron levels fall or H_2O_2 levels rise [11], the Fur or OxyR systems boost the rapid synthesis of MntH [5]. The consequent influx of manganese enables the activation of manganese-dependent isozymes of iron redox enzymes and probably the substitution of manganese for iron in mononuclear non-redox enzymes. Once manganese rises to an optimal level, MntR:Mn_2 forms and inhibits further MntH synthesis.

The problem with such an arrangement is that extant MntH will continue to import manganese—a situation that would especially overload the cell if extracellular manganese levels subsequently rise or if growth slows because of the absence of another nutrient. Continued manganese entry would also be undesirable if iron becomes available, flows into the cell, and is available to displace manganese from enzymes. A moderate excess of manganese would reduce the activity of mononuclear enzymes, which generally work better with iron [9,10], and a large excess would block heme synthesis and halt growth.

To avoid these outcomes bacteria containing excess manganese induce manganese exporters such as MntP. This balancing of metal importers against metal exporters is a common theme in transition-metal metabolism: over-action of the Zn importer ZnuABC is compensated for by induction of the Zn exporters ZitB and ZntA [55,56,57]; iron influx by the Feo, Fec, and Ent systems is poised against efflux by IceT and possibly FieF [58,59]; nickel influx by NikABC against efflux by RcnA [60]; and copper influx by unspecified systems against efflux by CopA [61].

Yet unfettered MntP activity runs into the same problem as unfettered MntH activity. If *E. coli* moves from a manganese-rich to a manganese-poor environment, new MntP synthesis will be turned down as MntR is deactivated—but the extant MntP will continue to pump manganese out of the cell. We suggest that MntS, whose synthesis is increased when manganese levels fall, may block this action of MntP. This 42-amino-acid protein might do so by directly binding and inhibiting MntP, in the way that the small protein SgrT is thought to block the action of glucose importers when sugar phosphates rise [62]. Our study has provided genetic data that fit this model; physical evidence awaits further work.

The multilayered system that we have proposed (Fig. 12) would operate in place of allosteric control. It would be much simpler if MntH turnover were inhibited when intracellular manganese levels rose and if MntP turnover were inhibited when those levels fell. By comparison, the magnesium importer MgtE features a cytoplasmic domain that gates its channel in response to the binding of multiple magnesium ions [63,64]. Why don't MntH and MntP work in this way? We speculate that such a system could be undermined by the theme that underlies all transition-metal metabolism: the difficulty that proteins have in distinguishing among these metals. Simple metal-binding sites are unlikely to discriminate between iron and manganese, for example. Investigators have shown that transcription factors require complex protein structures to specify their cognate metal [65]—and even so, MntR can bind iron and Fur can bind manganese [4]. Were allosteric sites on MntH to mistake iron for manganese, then the iron released from proteins during oxidative stress would prevent import of manganese, which would be exactly contrary to the purpose of the scheme. Perhaps the MntH/MntP/MntS system is the most successful arrangement that is structurally feasible and evolutionarily accessible.

Homology searches have only identified MntS in enteric bacteria that contain MntP [18]. The converse is not true: MntP is broadly distributed. It is possible that MntS has escaped detection in more-divergent organisms because its small size is problematic for homology-seeking programs; alternatively, either analogous proteins or mechanisms exist to control MntP in MntS-free bacteria, or else the circumstance does not arise in which MntP threatens to deplete

the cell of manganese. The lifestyles of enteric organisms involve transitions between anoxic and aerobic environments with profoundly different metal availabilities, which may necessitate the multiple stages of regulation to which MntS contributes.

Materials and Methods

Reagents

Antibiotics, 5-aminolevulinic acid hydrochloride (5-ALA), ß-mercaptoethanol, ß-NADH, bovine xanthine oxidase, casein acid hydrolysate, cytochrome *c* from equine heart, deamino-NADH, desferoxamine mesylate (DFO), diethylenetriamine pentaacetic acid (DTPA), 2,2'-dipyridyl (DIP), ethyl acetate, ferric chloride, ferrous ammonium sulfate, 30% hydrogen peroxide, 8-hydroxyquinoline-5-sulphonic acid, *E. coli* manganese-containing superoxide dismutase, manganese (II) chloride tetrahydrate, 2-[*N*-morpholino]ethanesulfonic acid (MES), *o*-dianisidine dihydrochloride, *o*-nitrophenyl-ß-D-galactopyranoside (ONPG), potassium ferricyanide, potassium cyanide, tricine, protoporphyrin IX, and xanthine were purchased from Sigma. Ethylenediamine tetraacetic acid (EDTA), guanidine hydrochloride, hydrochloric acid, and 3-(*N*-morpholino) propane-sulfonic acid (MOPS) were purchased from Fisher Scientific; Coomassie protein assay reagent and albumin standard, from Thermo Scientific; sodium dithionite, from Fluka; and glacial acetic acid, from J.T.Baker.

Bacterial strain and plasmid construction

The strains and plasmids that were used in this study are listed in S1 Table. Chromosomal null deletions were generated using the lambda Red recombination method [66]. All oxygen-sensitive strains were constructed under anoxic conditions to ensure that suppressor mutations were not selected during outgrowth. Mutations were introduced into new strains by P1 transduction [67]. All mutations were confirmed by PCR analysis or blue/white selection with Xgal. When necessary, the antibiotic cassette was removed by FLP-mediated excision [66]. The *mntS*-null deletion removes the entire gene sequence, including the non-coding region.

For single-copy *lacZ* transcriptional fusions, the promoter regions of given genes were amplified by PCR using primers designed with restriction sites. The product was digested and inserted into pSJ501, a plasmid derivative of pAH125 that was modified to express the chloramphenicol acetyl transferase gene (*cat*) flanked by FLP sites, thereby enabling selection under anoxic conditions. The resulting plasmid constructs were confirmed by restriction analysis and sequencing. Plasmids were then integrated into the λ attachment site, while the wild-type genes remained at their native positions [68]. Fusions were introduced into new strains by P1 transduction, and the chloramphenicol-resistance cassettes were removed by FLP-mediated excision [66].

The plasmid pMntS2 (pLW131) encodes the full 205 nt *rybA* transcript behind its own promoter. It was generated by amplifying 150 nt upstream of the *rybA* transcriptional start site, followed by 205 nt of the *rybA* sequence (including the complete MntS open reading frame) by PCR. The product was digested with SalI and XbaI and ligated into similarly digested pACYC184 (a low-copy-number plasmid that is maintained at ~15 copies/cell), which removes the tetracycline-resistance marker but maintains the chloramphenicol-resistance marker for selection.

The plasmid pMntS (pLW112) was generated by amplifying the MntS open reading frame and the upstream Shine-Dalgarno sequence by PCR [20]; other untranslated regions of the *rybA* transcript are not included. The product was digested with NheI and KpnI and cloned into pBAD24 (a medium-copy-number plasmid that is maintained at 15–20 copies/cell) behind the *araBAD* promoter.

The plasmids pMntS-F11 (pJEM67), pMntS-F16 (pJEMS68), pMntS-E3A (pMS017), pMntS-C7A (pMS018), pMntS-C27A (pMS020), pMntS-D28A (pMS021), and pMntS-H13A (pLW125), pMntS-E3A/C7A/D28A (pLW133), pMntS-E3A/C27A/D28A (pLW134), pMntS-C7A/C27A/D28A (pLW135), and pMntS-E3A/C7A/C27A (pLW136) express mutant *mntS* alleles that were created by site-directed mutagenesis on the template pLW122 using Pfu Turbo polymerase from Stratagene. Briefly, 60-base primers were designed with the mutation of interest located in the center of the sequence (S2 Table). Both forward and reverse complements were ordered. Mutagenesis was performed in a mixture (50 μl) containing 50 ng template DNA, 400 nM each primer complement, 200 μM dNTPs, and 2.5 units Pfu Turbo polymerase. Typical cycling conditions were as follows: 95°C/3 min; 18 cycles of 95°C/30 s, 55°C/1 min, 68°C/2.5 min/kb. The resulting mixture was digested with DpnI at 37°C for more than 1 hr to remove the original plasmid DNA template, and the mixture was then transformed into TOP10 electrocompetent *E. coli* cells, followed by selection on ampicillin plates. All resulting plasmids constructs were confirmed by sequencing.

Growth conditions

Luria broth (LB) and base M9 minimal salts were of standard composition [67]. Media were prepared using water that had been purified by a LabConco deionization system. Base MOPS minimal salts did not include $FeSO_4$ or micronutrients [69]. M9 and MOPS medium were supplemented with 0.2% glucose, 0.2% casamino acids, and 0.5 mM tryptophan (which is scant in casamino acids). When antibiotic selection was necessary, media were supplemented with 100 μg/ml ampicillin, 20 μg/ml chloramphenicol, 30 μg/ml kanamycin sulfate, or 12.5 μg/ml tetracycline hydrochloride. Anaerobic cultures were grown in an anaerobic chamber (Coy Laboratory Products Inc.) under an atmosphere of 85% N_2/10% H_2/5% CO_2. Aerobic cultures were grown under room air with vigorous shaking.

The manganese and iron content of the various media were measured by ICP-MS at the Center for Applied Isotope Studies of the University of Georgia. LB medium contained 7 μM iron and 200 nM manganese; MOPS glucose/amino acids medium contained 240 nM iron and 340 nM manganese; and M9 glucose/amino acids medium contained 800 nM iron and only 50 nM manganese.

To ensure that cells were growing exponentially, overnight cultures were diluted to OD_{600} 0.005 and grown at 37°C to an approximate OD_{600} of 0.12. Cells were then subcultured again into fresh aerobic medium to OD_{600} of 0.0025 and grown at 37°C to an approximate OD_{600} of 0.25 prior to analysis. Strains harboring pMntS or Δ*mntP* began exhibiting slower growth approximately 2 hr after incubation with manganese. Thus, enzyme activities were typically measured 2.5 hr after treatment with manganese.

Strains carrying pBAD24-derived plasmids were grown with ampicillin overnight and then diluted into fresh medium to OD_{600} of 0.005. After approximately four doublings, 50 mM L(+) arabinose was added to induce MntS expression. Cultures were grown for an additional 30 to 45 minutes, subcultured again to OD_{600} of 0.0025 in fresh aerobic medium containing ampicillin and 50 mM L(+)arabinose, and grown at 37°C.

Strains lacking *hemA* were grown overnight in anoxic LB medium and then diluted into fresh anoxic medium to OD_{600} of 0.005. After approximately three generations of growth, 0.25 mM 5-ALA was added to the medium. Cultures were grown for two additional generations, subcultured again to OD_{600} of 0.0025 using fresh aerobic medium containing 1 mM 5-ALA, and grown aerobically at 37°C.

Measurements of cell viability

Anaerobic overnight cultures in MOPS medium were diluted to OD_{600} of 0.005 in the same anaerobic medium and grown at 37°C to an OD_{600} of approximately 0.1. Cells were then subcultured again to OD_{600} of 0.0025 in fresh aerobic medium and grown at 37°C with vigorous shaking. At intervals, aliquots of cells were removed and serially diluted into aerobic medium. The diluted samples were transferred into the anaerobic chamber, mixed with anaerobic top agar, and poured onto anaerobic medium agar plates. Colonies were counted after 48 hours of anaerobic incubation at 37°C.

Enzyme assays

All enzyme assays were performed at room temperature. Protein concentrations were determined by the Coomassie assay according to the manufacturer's instructions, using bovine serum albumin as the standard.

ß-galactosidase activity. To prepare extracts, cells were centrifuged, washed twice, resuspended in 1/30 the original culture volume with ice-cold 50 mM Tris-HCl buffer (pH 8), and lysed by French press. Cell debris was removed by centrifugation, and the ß-galactosidase activity in cell extracts was determined by ONPG hydrolysis using standard procedures [67].

Superoxide dismutase activity. Mutants lacking *sodB* (encoding FeSOD) were used to track MnSOD activity. Extracts were prepared as for ß-galactosidase assays except that cells were resuspended in 1/100 the original culture volume. The SOD activity was measured in cell extracts using the xanthine oxidase/cytochrome *c* method [70]. After the initial assay, extracts were subjected to partial denaturation and renaturation in the presence of manganese to achieve full activation of MnSOD protein [11,71]. Briefly, MnSOD was denatured at pH 3.8 by dialysis against cold 5 mM Tris-HCl/2.5 M guanidinium chloride/20 mM 8-hydroxyquinoline-5-sulphonic acid/0.1 mM EDTA for approximately 12 hr in the dark. The inactive apoenzyme was then renatured by dialysis against cold 5 mM HEPES/0.1 mM $MnCl_2$ (pH 7.8) for two periods of approximately 12 hr each. Finally, excess metal was removed by dialysis at pH 7.8 against cold 5 mM Tris-HCl/0.1 mM EDTA for two periods of 4 hr each. The entire reconstitution process was performed at 4°C. The "% active MnSOD" reports the initial activity divided by the reconstituted activity. Since some fraction of protein does not survive the procedure, it is possible in some experiments for this number to exceed 100%. Purchased *E. coli* manganese-containing SOD was used as a control for the reconstitution procedure.

Hydroperoxidase I (KatG) activity. To prepare extracts, cells were washed twice with ice-cold 50 mM potassium phosphate buffer (pH 7.8), resuspended in 1/30 the original culture volume, and lysed by French press in ice-cold 10 mM potassium phosphate buffer (pH 6.4). Cell debris was removed by centrifugation, and HPI, the KatG catalase, was specifically assayed through its ability to act as a peroxidase. Extracts were added to 300 μM *o*-dianisidine and 900 μM H_2O_2 in 10 mM KPi (pH 6.4), and the oxidation of *o*-dianisidine was monitored at A_{460} [72].

NADH dehydrogenase I (Ndh1) activity. Cells were centrifuged and washed twice with ice-cold 50 mM MES buffer (pH 6.0). This pH protects the enzyme, which is unstable at higher pH. Final resuspension was in the same buffer at 1/60 the original culture volume. Cells were lysed by French press, and cell debris was removed by centrifugation. Inverted membrane vesicles were separated from the supernatant by ultra-centrifugation at 100,000 x *g* for 2 hr at 4°C. The vesicles were then resuspended in ice-cold 50 mM MES buffer (pH 6.0) at 1/120 the original culture volume. Vesicles were assayed immediately for NADH dehydrogenase activity at A_{340} with either 120 μM NADH or 60 μM deamino-NADH as the substrate in the same

room-temperature buffer. Ndh2 can use only NADH as a substrate, while Ndh1 can use both deamino-NADH and NADH with equal efficiency [73,74].

NADH dehydrogenase II (Ndh2) activity. Inverted membrane vesicles were isolated as described above. Resuspended inverted vesicles were diluted 5-fold into ice-cold 50 mM KPi (pH 7.8) and held at 0°C overnight to eliminate Ndh1 activity, which is unstable at this pH. The inverted vesicles were incubated in room temperature 50 mM MES buffer (pH 6) containing 3 mM KCN to block respiration through inhibition of cytochrome oxidase; the membranes were then assayed for Ndh2 activity by monitoring NADH oxidation at A_{340} in the presence of 200 μM $K_3Fe(CN)_6$, which acts as an oxidant that directly remove electrons from Ndh2.

Calculation of the fraction of cytoplasmic Mn that occupies MnSOD

Wild-type cells grown in LB medium contain ~ 12 U/mg total SOD activity, of which about one-third is conferred by MnSOD [75]. Since the total cytoplasmic protein concentration is 300 mg/ml, the cytoplasmic MnSOD activity is 1200 U/ml [76]. Fully Mn-loaded MnSOD exhibits a specific activity of 7300 U/mg [77], indicating that the concentration of Mn-loaded protein in the cytoplasm is 0.16 mg/ml. Since the subunit molecular weight is 23097 Daltons, this calculation indicates that the concentration of Mn-loaded subunits is ~7 μM. This compares to the measured total Mn concentration (Fig. 5A) of 5 μM. Some imprecision arises from multiple measurements to contribute to this calculation, but the implication is that under this growth condition most cytoplasmic Mn exists within MnSOD protein. A similar outcome was observed, using different methods, in *Bacillus anthracis* [78].

Electron paramagnetic resonance (EPR) measurements of unincorporated intracellular iron

The pool of intracellular chelatable iron was quantified by standard procedures [79] from one-liter cultures that were grown aerobically at 37°C in LB for 2.5 hr with or without 500 μM $MnCl_2$. Briefly, cells were centrifuged and resuspended at 1/100 the original culture volume in 37°C LB containing 10 mM DTPA (pH 7.0) to block further iron import and 20 mM DFO (pH 8.0) to facilitate the oxidation of intracellular unincorporated ferrous iron to EPR-detectable ferric iron. The cell mixture was incubated aerobically with vigorous shaking at 37°C for 15 min and then centrifuged. Cell pellets were washed twice with ice-cold 20 mM Tris-HCl/10% glycerol (pH 7.4) and finally resuspended in 150 μl of the same buffer. The final optical density was recorded after dilution of an aliquot, and samples were loaded into a quartz EPR tube. Samples were frozen on dry ice and stored at -80°C for no longer than one week. EPR standards consisted of $FeCl_3$ dissolved in 20 mM Tris-HCl/10% glycerol/1 mM DFO (pH 7.4); the iron concentration in the standard was determined using $€_{mM}$ at 420 nM of 2.865 cm^{-1}. EPR spectra were acquired on a Varian Century E-112 X-band spectrophotometer at 15 K using a Varian TE102 cavity using 10 mW power, 12.5 G modulation amplitude, 4000 gain, 32 ms time constant, and 100 kHz modulation frequency. EPR spectra for samples were normalized to cell density and converted to intracellular iron concentrations using the following conversion: 1 ml of bacteria culture at 1 OD_{600} equals 0.52 μl of intracellular volume [76].

Detection of manganese by EPR

Reduced manganese (Mn^{2+}) spectra were detected from one-liter cultures of JEM1280 and JEM1281 that were grown aerobically at 37°C in LB for 2.5 hr with or without 500 μM $MnCl_2$. IPTG (1 mM) was added when cultures reached OD_{600} ~ 0.2, and cells were harvested at OD_{600} ~ 0.4. Cells were washed twice and resuspended in 1/2000 the original culture volume with ice-cold 100 mM Tris-HCl/150 mM NaCl/5% glycerol (pH 7.6). Samples were adjusted to

similar densities, approximately 110 OD_{600}, and loaded into quartz EPR tubes. Samples were frozen on dry ice and stored at -80°C for no longer than one week. EPR spectra were acquired at 110 K using 2 mW power, 5 G modulation amplitude, 20000 gain, 32 ms time constant, and 100 kHz modulation frequency.

Inductively coupled plasma-mass spectrometry (ICP-MS) of intracellular iron and manganese

The total amounts of intracellular iron and manganese were quantified from one-liter cultures that had been grown aerobically at 37°C in LB for 2.5 hr with or without 0.5 mM $MnCl_2$. Cells were centrifuged and washed twice with ice-cold 20 mM Tris-HCl/1 mM EDTA (pH 7.4) and once with ice-cold 20 mM Tris-HCl (pH 7.4). Cells were then resuspended in ice cold 20 mM Tris-HCl (pH 7.4) to 1/500 the original culture volume and lysed by French press. Cell debris was removed by centrifugation. The metal content was determined at the University of Georgia Center for Applied Isotope Studies and normalized to total protein in the lysates. Intracellular concentrations were calculated based on ~300 mg/ml intracellular protein concentration, which was derived from measurements of cell volume (Imlay and Fridovich, 1991) and of 175 mg soluble protein harvested per L-OD of *E. coli*.

Porphyrin quantification

Porphyrins were extracted from 100 ml cultures that were grown aerobically at 37°C in LB/arabinose medium for 2.5 hr with or without 500 μM $MnCl_2$ [80]. Cells were centrifuged, washed twice with ice-cold 50 mM Tris-HCl (pH 8.0), resuspended in ethyl acetate/glacial acetic acid (3:1, v/v) to 1/100 the original culture volume, and lysed by sonication on ice. Cell debris was removed by centrifugation at room temperature, and the non-aqueous (top) phase was washed twice with 1 ml ddH_2O to remove residual water-soluble contaminants, taking care not to disturb the intermediate phase. Porphyrins were then extracted from the solution by the addition of 0.5 ml 3 M HCl, and the absorbance of the aqueous (bottom) phase was assessed at 408 nm. Porphyrin levels were normalized to optical cell density (OD_{600}).

Quantification of intracellular protoporphyrin IX was performed upon 500 ml cultures that had been grown in aerobic LB medium for 2.5 hr with or without 500 μM $MnCl_2$. Cells were centrifuged, washed with ice-cold 50 mM Tris-HCl (pH 8.0), and normalized to similar densities, approximately 15 OD_{600} in 1/250 the original culture volume. Samples were frozen on dry ice/ethanol bath and stored at -80°C for no longer than one week. Porphyrins were extracted from thawed cells the day of LC/MS/MS analysis as described above with the addition of 0.1 ml 3 M HCl for the last step in the procedure. Samples were analyzed with the Metabolomics Center 5500 QTRAP LC/MS/MS system (AB Sciex, Foster City, CA) with a 1200 series HPLC system (Agilent Technologies, Santa Clara, CA) including a degasser, an autosampler, and a binary pump. The LC separation was performed on an Agilent SB-Aq column (4.6 x 50 mm, 5 μm) (Santa Clara, CA) with mobile phase A (0.1% formic acid in water) and mobile phase B (0.1% formic acid in acetontrile). The flow rate was 0.3 mL/min. The linear gradient was as follows: 0–1 min, 100%A; 10–18 min, 5%A; 19–24 min, 100% A. The autosampler was set at 5°C. The injection volume was 1 μL. Mass spectra were acquired with positive electrospray ionization (ESI) and the ion spray voltage was 5500 V. The source temperature was 450°C. The curtain gas, ion source gas 1, and ion source gas 2 were 32, 65, and 50, respectively. Multiple reaction monitoring (MRM) was used to monitor protoporphyrin IX (m/z 563.2 —> m/z 504.1) using an authentic standard obtained from Sigma.

Western blot analysis of MntP protein

Strain MS033 includes the SPA tag inserted into the chromosome fused to the *mntP* ORF. Therefore, the fusion protein is expressed from the native *mntP* promoter and the native 5' UTR leaving both MntR- and riboswitch-mediated regulation are intact. Cultures of MS033 containing either pBAD24 or pMntS were grown overnight in M9 medium with 0.2% glucose and ampicillin, diluted 1:100 into fresh medium, and grown to OD_{600} ~0.2. Cells were washed twice with M9 medium lacking a carbon source and resuspended in M9 medium containing 0.2% arabinose, in order to induce MntS synthesis. Cells were grown for 10 min at 37°C, washed twice with M9 medium lacking a carbon source, and resuspended in M9 medium with 0.2% glucose, ampicillin, and 10 mM $MnCl_2$ in order to induce MntP synthesis. Time points were taken as indicated. For western blot analysis, cells were lysed by resuspension in 1x SDS loading buffer with 100 mM dithiothreitol and heated at 95°C for 10 min. Whole-cell lysate corresponding to ~0.03 OD_{600} units of cells was separated on 4–20% Tris-Glycine gels (Bio-Rad) and transferred to nitrocellulose membranes (Bio-Rad). Membranes were blocked with 2% milk in Tris-buffered saline with Tween (TBS-T) and probed with anti-FLAG M2-AP antibody (Sigma-Aldrich) in 2% milk–TBS-T. Signals were visualized using Lumi-PhosWB (Pierce).

Supporting Information

S1 Fig. Complementation of the growth defect of the Δ*mntS* mutant. Cells were pre-cultured in anaerobic M9 glucose/casamino acids medium and then diluted at time zero into the same aerobic medium containing 15 μM H_2O_2. Strains used were LC106 (Hpx^-) and JEM1177 (Hpx^- Δ*mntS*) expressing pMntS2 (pLW131, *mntS* under its own promoter). The data are representative of at least three independent experiments.
(TIF)

S2 Fig. MntH function does not require the presence of MntR or MntH. Cells were pre-cultured in anaerobic M9 glucose/casamino acids medium and then diluted at time zero into the same aerobic medium with or without 15 μM H_2O_2. The data are representative of at least three independent experiments. A. MntS functions in the absence of *mntR*. Strains used were JEM1216 [Hpx^- Δ(*mntS-mntR*)] carrying an empty vector (pACYC184) or pMntS2 (pLW131, *mntS* under its own promoter). B. MntH functions in the absence of *mntS*. Strains were JEM1177 (Hpx^- Δ*mntS*) and JEM1227 (Hpx^- Δ*mntS* Δ*mntH*). C. MntS functions in the absence of *mntH*. OD_{600} of strains AA30 (Hpx^- Δ*mntH*) and JEM1227 (Hpx^- Δ*mntH* Δ*mntS*) grown in the presence of increasing concentrations of $MnCl_2$ for 6 hr.
(TIF)

S3 Fig. Whole-cell EPR spectra of manganese. Cultures were grown with 500 μM manganese in LB medium. Peaks represent Mn^{2+}. EPR peak heights varied with point of harvest and do not provide a precise comparison of Mn content. Temperature = 30 K, modulation = 5 g, power = 2 mW.
(TIF)

S4 Fig. The Δ*fur* mutation suppresses the pMntS growth defect even in a Δ*ryhB* background. Cells were pre-cultured in aerobic LB medium and then diluted at time zero into fresh LB/arabinose medium with 0.5 mM $MnCl_2$. Strains were JEM1542/JEM1536 (Δ*ryhB*) harboring empty vector (pBAD24) or pMntS (pLW112, *mntS* driven by the *araBAD* promoter) and JEM1538/JEM1540 (Δ*fur* Δ*ryhB*) harboring empty vector or pMntS. The data are

representative of at least three independent experiments.
(TIF)

S5 Fig. Manganese does not impede iron-sulfur cluster assembly. Cells were grown in anaerobic LB/arabinose medium with or without 0.5 mM $MnCl_2$ and aerated for 2.5 hr before harvesting. Data represent the mean of three independent cultures. A, B. Levels of NADH dehydrogenase 1 (an iron-sulfur enzyme) and NADH dehydrogenase 2 (an iron-free enzyme) are not diminished during manganese intoxication. Strains were OD502 (Δ*suf*) harboring empty vector (pBAD24) or pMntS (pLW112, *mntS* driven by the *araBAD* promoter). C. Transcription of the *iscR* and *sufA* genes was not induced during manganese intoxication, indicating that IscR remained in its cluster-containing form. Strains bearing *iscR'-lacZ* were JEM1474 (WT/pBAD24) and JEM1475 (WT/pLW112). Strains bearing *sufA'-lacZ* were JEM1476 (WT/pBAD24) and JEM1477 (WT/pLW112).
(TIF)

S6 Fig. Low catalase G activity does not result from diminished transcription of *katG*. Cells bearing the *katG'-lacZ* transcriptional fusion were pre-cultured in aerobic LB and then diluted into LB/arabinose medium with or without 0.5 mM $MnCl_2$. Cells were harvested after 2.5 hr treatment. Strains were AL441 harboring empty vector (pBAD24) or pMntS (pLW112).
(TIF)

S7 Fig. Supplementation with 5-ALA does not suppress manganese sensitivity in Δ*hemA* mutants overexpressing MntS. A. The heme biosynthentic pathway. B. Cells lacking *hemA* were pre-cultured in anaerobic LB medium and then diluted at time zero into fresh aerobic LB/arabinose medium with or without 5-ALA. Strains were SMA1091 (Δ*hemA*) harboring empty vector (pBAD24) or pMntS (pLW112, *mntS* driven by the *araBAD* promoter). The data are representative of at least three independent experiments.
(TIF)

S8 Fig. Heme synthesis is inhibited by MntS overproduction. *hemA*-null mutants were grown in aerobic LB/arabinose medium supplemented with 1 mM 5-ALA. Cells were harvested and intracellular porphyrins were quantified after 2.5 hr treatment with or without 0.5 mM $MnCl_2$ or 100 μM DIP. Data represent the mean of three independent cultures. Strains were JEM1579 (Δ*hemA*/pBAD24), JEM1580 (Δ*hemA*/pLW112), JEM1683 (Δ*mntP*/pBAD24).
(TIF)

S9 Fig. The withdrawal of essential cofactors does not cause an immediate block in growth. (A) The wild-type strain MG1655 and mutant strains defective in synthesis of thiamine (AB1157), lipoic acid (KER176), and biotin (NRD25) were cultured for 4 generations in minimal A medium supplemented with 0.5 mM of the 20 standard amino acids, plus 5 μg/ml of the required vitamin. At time zero the cells were then centrifuged, washed three times, and suspended in the same media lacking vitamins. Growth was monitored by absorbance. Cells were repeatedly subcultured to maintain densities < 0.3 OD_{600}, and data are presented as the amount of residual growth after removal of the vitamins. (B) A Δ*hemA* derivative of MG1655 (SMA1139) was cultured $>$ 4 generations in aerobic minimal A medium containing 1% casamino acids as the sole carbon source plus 1 mM 5-aminolevulinic acid (ALA) to enable heme synthesis. The medium was chosen to mimic the effects of LB medium while avoiding the presence of peptides, since ALA is imported through the dipeptide transporter. At time zero the exponentially growing cells were centrifuged, washed three times, and suspended in the same medium +/- ALA, and growth was monitored.
(TIF)

S10 Fig. The combination of MntS overexpression and Mn supplementation do not impair anaerobic growth. MG1655 strains containing an empty vector (pBAD24; squares) or containing pMntS (pLW112; triangles) were grown exponentially in anoxic LB medium. At time zero cells were diluted into the same medium with no additions (filled symbols) or with 0.5 mM $MnCl_2$ (open symbols), and subsequent growth was monitored. The analogous experiment under aerobic conditions causes complete growth arrest for the Mn-supplemented pMntS strain (Figs 4, 9).
(TIF)

S11 Fig. Cells were grown in anaerobic LB/arabinose medium with or without 0.5 mM $MnCl_2$ and aerated for 2.5 hr before harvesting. Data represent the mean of three independent cultures. A. Transcription levels of the Fur-regulated genes *iucC'-lacZ* and *fhuA'-lacZ* in WT strains (JEM271 and GS45, repectively) harboring the indicated plasmids. B. KatG activity determined from WT (GS45) strains harboring the indicated plasmids.
(TIF)

S12 Fig. MntS does not speed the metallation of MnSOD in vitro. Cell extracts were prepared from the Δ*sodB ΔmntS* strain JEM1234 containing pDT1–16, which overexpresses *sodA*. Extract were treated to remove Mn from the MnSOD protein. Remetallation was performed in 37° C pH 7.8 Tris/EDTA buffer through the addition of 200 μM $MnCl_2$, with or without the addition of 0.36 μM purified MntS. The graph has been scaled to emphasize the early time; two- and three-hour time points revealed the same activity as the one-hour time point.
(TIF)

S13 Fig. MntS overproduction does not worsen the metal dys-homeostasis of Δ*mntP* mutants. Cells pre-cultured in aerobic LB medium were diluted into fresh LB/arabinose medium with or without 0.5 mM $MnCl_2$ and harvested after 2.5 hr of aerobic growth, followed by ICP-MS analysis (A and B) or porphyrin accumulation (C). Data represent the mean of three independent cultures. Strains were MS025 (Δ*mntP*) harboring empty vector (pBAD24) or pMntS (pLW112, *mntS* driven by the *araBAD* promoter). Note that data represented by empty vector has been reprinted from Fig. 10 to aid data comparison.
(TIF)

S14 Fig. Overexpression of *mntS* does not inhibit MntP accumulation. Cultures were grown in M9 glucose medium + ampicillin to 0.2 OD_{600}. Cells were washed and resuspended in arabinose medium for 10 min to induce MntS. They were then washed again and suspended in the original glucose medium supplemented with 10 μM manganese to induce MntP from its native promoter and to reproduce the growth phenotype. At intervals cells were harvested and MntP-SPA content was evaluated by western blot with anti-SPA antibodies. The higher MntP levels in the *mntS*-overexpressing strains likely results from the increased manganese levels under these conditions (Fig. 5A), since MntP synthesis is induced by manganese [20].
(TIF)

S15 Fig. Excess manganese blocks *ryhB* expression by metallating Fur protein. The *ryhB-'lacZ* transcriptional fusion strain JEM1500 (with vector), JEM1501 (pMntS), JEM1503 (*fur* with pBAD vector) and JEM1504 (*fur* with pMntS) were grown exponentially in aerobic LB/arabinose medium, and ß-galactosidase activity was assayed as a representation of *ryhB* expression. Where indicated dipyridyl (0.1 mM) was added to moderately restrict iron availability, or an inhibitory dose of manganese (0.5 mM) was added to toxify the pMntS strain. Error bars indicate the standard deviation from 3–4 replicates.
(TIF)

S1 Table. Strains and plasmids.
(DOCX)

S2 Table. Primers used in site-directed mutagenesis.
(DOCX)

S3 Table. Heme proteins of *Escherichia coli* K-12. Non-K-12 strains additionally express heme-containing soluble cytochrome b_{562} (CybC) and ferrous iron transporter (EfeUOB), both of which are cryptic in K-12 strains. *E. coli* K-12 synthesizes two proteins with siroheme cofactors: sulfite reductase and soluble nitrite reductase (NirB). Ferrochelatase is not involved in siroheme synthesis.
(DOCX)

Acknowledgments

We thank Stefano Mancini and Zhong Li for help with porphyrin measurements, Mark Nilges for his assistance with EPR analyses, Melissa Sandoval for her help with plasmid constructions, and Kari Imlay and Amy Wu for performing supplementary control experiments. Dr. Mancini provided the information in S3 Table.

Author Contributions

Conceived and designed the experiments: JAI JEM GS LSW. Performed the experiments: JEM LSW. Analyzed the data: JEM LSW GS JAI. Contributed reagents/materials/analysis tools: LSW GS JAI. Wrote the paper: JEM LSW GS JAI.

References

1. Anbar AD (2008) Elements and evolution. Science 322: 1481–1483. doi: 10.1126/science.1163100 PMID: 19056967
2. Andrews SC, Robinson AK, Rodriguez-Quinones F (2003) Bacterial iron homeostasis. FEMS Microbiol Rev 27: 215–237. PMID: 12829269
3. Masse E, Gottesman S (2002) A small RNA regulates the expression of genes involved in iron metabolism in *Escherichia coli*. Proc Natl Acad Sci U S A 99: 4620–4625. PMID: 11917098
4. Ikeda J, Janakiraman A, Kehres DG, Maguire ME, Slauch JM (2005) Transcriptional regulation of *sitABCD* of *Salmonella enterica* serovar Typhimurium by MntR and Fur. J Bacteriol 187: 912–922. PMID: 15659669
5. Kehres DG, Janakiraman A, Slauch JM, Maguire ME (2002) Regulation of *Salmonella enterica* serovar Typhimurium *mntH* transcription by H_2O_2, Fe^{2+}, and Mn^{2+}. J Bacteriol 184: 3151–3158. PMID: 12029030
6. Compan I, Touati D (1993) Interaction of six global transcription regulators in expression of manganese superoxide dismutase in *Escherichia coli* K-12. J Bacteriol 175: 1687–1696. PMID: 8449876
7. Cotruvo JA, Stubbe J (2011) *Escherichia coli* class Ib ribonucleotide reductase contains a dimanganese(III)-tyrosyl radical cofactor in vivo. Biochemistry 50: 1672–1681. doi: 10.1021/bi101881d PMID: 21250660
8. Martin JE, Imlay JA (2011) The alternative aerobic ribonucleotide reductase of *Escherichia coli*, NrdEF, is a manganese-dependent enzyme that enables cell replication during periods of iron starvation. Mol Microbiol 80: 319–334. doi: 10.1111/j.1365-2958.2011.07593.x PMID: 21338418
9. Anjem A, Imlay JA (2012) Mononuclear iron enzymes are primary targets of hydrogen peroxide stress. J Biol Chem 287: 15544–15556. doi: 10.1074/jbc.M111.330365 PMID: 22411989
10. Sobota JM, Imlay JA (2011) Iron enzyme ribulose-5-phosphate 3-epimerase in *Escherichia coli* is rapidly damaged by hydrogen peroxide but can be protected by manganese. Proc Natl Acad Sci USA 108: 5402–5407. doi: 10.1073/pnas.1100410108 PMID: 21402925
11. Anjem A, Varghese S, Imlay JA (2009) Manganese import is a key element of the OxyR response to hydrogen peroxide in *Escherichia coli*. Mol Microbiol 72: 844–858. doi: 10.1111/j.1365-2958.2009.06699.x PMID: 19400769

12. Kehres DG, Zaharik ML, Finlay BB, Maguire ME (2000) The NRAMP proteins of *Salmonella typhimurium* and *Escherichia coli* are selective manganese transporters involved in the response to reactive oxygen. Mol Microbiol 36: 1085–1100. PMID: 10844693

13. Grass G, Wong MD, Rosen BP, Smith RL, Rensing C (2002) ZupT is a Zn(II) uptake system in *Escherichia coli*. J Bacteriol 184: 864–866. PMID: 11790762

14. Silver S, Johnseine P, Whitney E, Clark D (1972) Manganese-resistant mutants of *Escherichia coli*: physiological and genetic studies. J Bacteriol 110: 186–195. PMID: 4552988

15. Hantke K (1987) Selection procedure for deregulated iron transport mutants (*fur*) in *Escherichia coli* K 12: *fur* not only affects iron metabolism. Mol Gen Genet 0: 135–139.

16. Glasfeld A, Guedon E, Helmann JD, Brennan RG (2003) Structure of the manganese-bound manganese transport regulator of *Bacillus subtilis*. Nat Struct Biol 10: 652–657. PMID: 12847518

17. Patzer SI, Hantke K (2001) Dual repression by Fe^{2+}-Fur and Mn^{2+}-MntR of the *mntH* gene, encoding an NRAMP-like Mn^{2+} transporter in *Escherichia coli*. J Bacteriol 183: 4806–4813. PMID: 11466284

18. Rosch JW, Gao G, Ridout G, Wang YD, Tuomanen EI (2009) Role of the manganese efflux system *mntE* for signalling and pathogenesis in *Streptococcus pneumoniae*. Mol Microbiol 72: 12–25. doi: 10.1111/j.1365-2958.2009.06638.x PMID: 19226324

19. Veyrier FJ, Boneca IG, Cellier MF, Taha MK (2011) A novel metal transporter mediating manganese export (MntX) regulates the Mn to Fe intracellular ratio and *Neisseria meningitidis* virulence. PLoS Pathog 7: e1002261. doi: 10.1371/journal.ppat.1002261 PMID: 21980287

20. Waters LS, Sandoval M, Storz G (2011) The *Escherichia coli* MntR miniregulon includes genes encoding a small protein and an efflux pump required for manganese homeostasis. J Bacteriol 193: 5887–5897. doi: 10.1128/JB.05872-11 PMID: 21908668

21. Seaver LC, Imlay JA (2001) Alkyl hydroperoxide reductase is the primary scavenger of endogenous hydrogen peroxide in *Escherichia coli*. J Bacteriol 183: 7173–7181. PMID: 11717276

22. Keele BB Jr., McCord JM, Fridovich I (1970) Superoxide dismutase from *Escherichia coli* B. A new manganese-containing enzyme. J Biol Chem 245: 6176–6181. PMID: 4921969

23. Whittaker MM, Whittaker JW (1997) Mutagenesis of a proton linkage pathway in *Escherichia coli* manganese superoxide dismutase. Biochemistry 36: 8923–8931. PMID: 9220980

24. Ikeda J, Janakiraman A, Kehres DG, Maguire ME, Slauch JM (2005) Transcriptional regulation of *citABCD* of *Salmonella enterica* serovar Typhimurium by MntR and Fur. J Bacteriol 187: 912–922. PMID: 15659669

25. Taudte N, Grass G (2010) Point mutations change specificity and kinetics of metal uptake by ZupT from *Escherichia coli*. Biometals 23: 643–656. doi: 10.1007/s10534-010-9319-z PMID: 20225068

26. van Veen HW, Abee T, Kortstee GJ, Konings WN, Zehnder AJ (1994) Translocation of metal phosphate via the phosphate inorganic transport system of *Escherichia coli*. Biochemistry 33: 1766–1770. PMID: 8110778

27. Hamed MY (1993) Binding of the ferric uptake regulation repressor protein (Fur) to Mn(II), Fe(II), Co(II), and Cu(II) ions as co-repressors: electronic absorption, equilibrium, and ^{57}Fe Mossbauer studies. J Inorg Biochem 50: 193–210. PMID: 8501465

28. Py B, Barras F (2010) Building Fe-S proteins: bacterial strategies. Nat Rev Microbiol 8: 436–446. doi: 10.1038/nrmicro2356 PMID: 20467446

29. Jang S, Imlay JA (2010) Hydrogen peroxide inactivates the *Escherichia coli* Isc iron-sulphur assembly system, and OxyR induces the Suf system to compensate. Mol Microbiol 78: 1448–1467. doi: 10.1111/j.1365-2958.2010.07418.x PMID: 21143317

30. Schwartz CJ, Djaman O, Imlay JA, Kiley PJ (2000) The cysteine desulfurase, IscS, has a major role in *in vivo* Fe-S cluster formation in *Escherichia coli*. Proc Natl Acad Sci USA 97: 9009–9014. PMID: 10908675

31. Nesbit AD, Giel JL, Rose JC, Kiley PJ (2009) Sequence-specific binding to a subset of IscR-regulated promoters does not require IscR Fe-S cluster ligation. J Mol Biol 387: 28–41. doi: 10.1016/j.jmb.2009.01.055 PMID: 19361432

32. Baba T, Ara T, Hasegawa M, Takai Y, Okumura Y, et al. (2006) Construction of *Escherichia coli* K-12 in-frame, single-gene knockout mutants: the Keio collection. Mol Syst Biol 2: 2006.0008. PMID: 16738554

33. Wassarman KM, Repoila F, Rosenow C, Storz G, Gottesman S (2001) Identification of novel small RNAs using comparative genomics and microarrays. Genes Dev 15: 1637–1651. PMID: 11445539

34. Gerstle K, Klatschke K, Hahn U, Piganeau N (2012) The small RNA RybA regulates key genes in the biosynthesis of aromatic amino acids under peroxide stress in *E*. *coli*. RNA Biol 9: 458–468. doi: 10.4161/rna.19065 PMID: 22336764

35. De Lay N, Schu DJ, Gottesman S (2013) Bacterial small RNA-based negative regulation: Hfq and its accomplices. J Biol Chem 288: 7996–8003. doi: 10.1074/jbc.R112.441386 PMID: 23362267

36. Imlay JA, Chin SM, Linn S (1988) Toxic DNA damage by hydrogen peroxide through the Fenton reaction in vivo and in vitro. Science 240: 640–642. PMID: 2834821

37. Touati D, Jacques M, Tardat B, Bouchard L, Despied S (1995) Lethal oxidative damage and mutagenesis are generated by iron in delta *fur* mutants of *Escherichia coli*: protective role of superoxide dismutase. J Bacteriol 177: 2305–2314. PMID: 7730258

38. Macomber L, Imlay JA (2009) The iron-sulfur clusters of dehydratases are primary intracellular targets of copper toxicity. Proc Natl Acad Sci USA 106: 8344–8349. doi: 10.1073/pnas.0812808106 PMID: 19416816

39. Azzouzi A, Steunou A-S, Durand A, Khalfaoui-Hassani B, Bourbon M-L, et al. (2013) Coproporphyrin III excretion identifies the anaerobic coproporphyrinogen III oxidase HemN as a copper target in the Cu^{+}-ATPase mutant *copA*⁻ of *Rubrivivax gelatinosus*. Mol Microbiol 88: 339–351. doi: 10.1111/mmi.12188 PMID: 23448658

40. Gu M, Imlay JA (2013) Superoxide poisons mononuclear iron enzymes by causing mismetallation. Mol Microbiol 89: 123–134. doi: 10.1111/mmi.12263 PMID: 23678969

41. Barras F, Fontecave M (2011) Cobalt stress in *Escherichia coli* and *Salmonella enterica*: molecular bases for toxicity and resistance. Metallomics 3: 1130–1134. doi: 10.1039/c1mt00099c PMID: 21952637

42. Macomber L, Elsey SP, Hausinger RP (2011) Fructose-1,6-bisphosphate aldolase (class II) is the primary site of nickel toxicity in *Escherichia coli*. Mol Microbiol 82: 1291–1300. doi: 10.1111/j.1365-2958.2011.07891.x PMID: 22014167

43. Macomber L, Hausinger RP (2011) Mechanisms of nickel toxicity in microorganisms. Metallomics 3: 1153–1162. doi: 10.1039/c1mt00063b PMID: 21799955

44. Dailey HA (1987) Metal inhibition of ferrochelatase. Ann N Y Acad Sci 514: 81–86. PMID: 3442391

45. Medlock AE, Carter M, Dailey TA, Dailey HA, Lanzilotta WN (2009) Product release rather than chelation determines metal specificity for ferrochelatase. J Mol Biol 393: 308–319. doi: 10.1016/j.jmb.2009.08.042 PMID: 19703464

46. Irving H, Williams RJP (1948) Order of stability of metal complexes. Nature 162: 746–747.

47. Masse E, Gottesman S (2002) A small RNA regulates the expression of genes involved in iron metabolism in *Escherichia coli*. Proc Natl Acad Sci USA 99: 4620–4625. PMID: 11917098

48. Salvail H, Lanthier-Bourbonnais P, Sobota JM, Caza M, Benjamin JA, et al. (2010) A small RNA promotes siderophore production through transcriptional and metabolic remodeling. Proc Natl Acad Sci U S A 107: 15223–15228. doi: 10.1073/pnas.1007805107 PMID: 20696910

49. Hohle TH, O'Brian MR (2012) Manganese is required for oxidative metabolism in unstressed *Bradyrhizobium japonicum* cells. Mol Microbiol 84: 766–777. doi: 10.1111/j.1365-2958.2012.08057.x PMID: 22463793

50. Yang J, Sangwan I, Lindemann A, Hauser F, Hennecke H, et al. (2006) *Bradyrhizobium japonicum* senses iron through the status of haem to regulate iron homeostasis and metabolism. Mol Microbiol 60: 427–437. PMID: 16573691

51. Puri S, Hohle TH, O'Brian MR (2010) Control of bacterial iron homeostasis by manganese. Proc Natl Acad Sci USA 107: 10691–10695. doi: 10.1073/pnas.1002342107 PMID: 20498065

52. Archibald F (1986) Manganese: its acquisition by and function in the lactic acid bacteria. Crit Rev Microbiol 13: 63–109. PMID: 3522109

53. Daly MJ, Gaidamakova EK, Matrosova VY, Valilenko A, Zhai M, et al. (2004) Accumulation of Mn(II) in *Deinococcus radiodurans* facilitates gamma-radiation resistance. Science 306: 1025–1028. PMID: 15459345

54. Baureder M, Hederstedt L (2013) Heme proteins in lactic acid bacteria. Adv Microb Physiol 62: 1–43. doi: 10.1016/B978-0-12-410515-7.00001-9 PMID: 23481334

55. Rensing C, Mitra B, Rosen BP (1997) The *zntA* gene of *Escherichia coli* encodes a Zn(II)-translocating P-type ATPase. Proc Natl Acad Sci USA 94: 14326–14331. PMID: 9405611

56. Grass G, Fan B, Rosen BP, Franke S, Nies DH, et al. (2001) ZitB (YbgR), a member of the cation diffusion facilitator family, is an additional zinc transporter in *Escherichia coli*. J Bacteriol 183: 4664–4667. PMID: 11443104

57. Wang D, Hosteen O, Fierke CA (2012) ZntR-mediated transcription of *zntA* responds to nanomolar intracellular free zinc. J Inorg Biochem 111: 173–181. doi: 10.1016/j.jinorgbio.2012.02.008 PMID: 22459916

58. Frawley ER, Crouch M-LV, Bingham-Ramos LK, Robbins HF, Wang W, et al. (2013) Iron and citrate export by a major facilitator superfamily pump regulates metabolism and stress resistance in *Salmonella* typhimurium. Proc Natl Acad Sci USA 110: 12054–12059. doi: 10.1073/pnas.1218274110 PMID: 23821749

59. Grass G, Otto M, Fricke B, Haney CJ, Rensing C, et al. (2005) FieF (YiiP) from *Escherichia coli* mediates decreased cellular accumulation of iron and relieves iron stress. Arch Microbiol 183: 9–18. PMID: 15549269

60. Rorigue A, Effantin G, Mandrand-Berthelot MA (2005) Identification of *rcnA* (*yohM*), a nickel and cobalt resistance gene in *Escherichia coli*. J Bacteriol 187: 2912–2916. PMID: 15805538

61. Rensing C, Fan B, Sharma R, Mitra B, Rosen BP (2000) CopA: an *Escherichia coli* Cu(I)-translocating P-type ATPase. Proc Natl Acad Sci U S A 97: 652–656. PMID: 10639134

62. Vanderpool CK, Balasubramanian D, Lloyd CR (2011) Dual-function RNA regulators in bacteria. Biochemie 93: 1943–1949.

63. Hattori M, Tanaka Y, Fukai S, Ishitani R, Nureki O (2007) Crystal structure of the MgtE Mg^{2+} transporter. Nature 448: 1072–1076. PMID: 17700703

64. Hattori M, Iwase N, Furuya N, Tanaka Y, Tsukazaki T, et al. (2009) Mg^{2+}-dependent gating of bacterial MgtE channel underlies Mg^{2+} homeostasis. EMBO J 28: 3602–3612. doi: 10.1038/emboj.2009.288 PMID: 19798051

65. Ma Z, Jacobsen FE, Giedroc DP (2009) Coordination chemistry of bacterial metal transport and sensing. Chem Rev 109: 4644–4681. doi: 10.1021/cr900077w PMID: 19788177

66. Datsenko KA, Wanner BL (2000) One-step inactivation of chromosomal genes in *Escherichia coli* K-12 using PCR products. Proc Natl Acad Sci U S A 97: 6640–6645. PMID: 10829079

67. Miller JH (1972) Experiments in Molecular Genetics. Cold Spring Harbor, N.Y.: Cold Spring Harbor Laboratory.

68. Haldimann A, Wanner BL (2001) Conditional-replication, integration, excision, and retrieval plasmid-host systems for gene structure-function studies of bacteria. J Bacteriol 183: 6384–6393. PMID: 11591683

69. Neidhardt FC, Bloch PL, Smith DF (1974) Culture medium for enterobacteria. J Bacteriol 119: 736–747. PMID: 4604283

70. McCord JM, Fridovich I (1969) Superoxide dismutase. An enzymic function for erythrocuprein (hemocuprein). J Biol Chem 244: 6049–6055. PMID: 5389100

71. Kirby T, Blum J, Kahane I, Fridovich I (1980) Distinguishing between Mn-containing and Fe-containing superoxide dismutases in crude extracts of cells. Arch Biochem Biophys 201: 551–555. PMID: 6994652

72. Macomber L, Rensing C, Imlay JA (2007) Intracellular copper does not catalyze the formation of oxidative DNA damage in *Escherichia coli*. J Bacteriol 189: 1616–1626. PMID: 17189367

73. Calhoun MW, Gennis RB (1993) Demonstration of separate genetic loci encoding distinct membrane-bound respiratory NADH dehydrogenases in *Escherichia coli*. JBact 175: 3013–3019. PMID: 8387992

74. Matsushita K, Ohnishi T, Kaback HR (1987) NADH-ubiquinone oxidoreductases of the *Escherichia coli* aerobic respiratory chain. Biochemistry 26: 7732–7737. PMID: 3122832

75. Varghese S, Wu A, Park S, Imlay KRC, Imlay JA (2007) Submicromolar hydrogen peroxide disrupts the ability of Fur protein to control free-iron levels in *Escherichia coli*. Mol Microbiol 64: 822–830. PMID: 17462026

76. Imlay JA, Fridovich I (1991) Assay of metabolic superoxide production in *Escherichia coli*. J Biol Chem 266: 6957–6965. PMID: 1849898

77. Edwards RA, Whittaker MM, Whittaker JW, Baker EN, Jameson GB (2001) Outer sphere mutations perturb metal reactivity in manganese superoxide dismutase. Biochemistry 40: 15–27. PMID: 11141052

78. Tu WY, Pohl S, Gray J, Robinson NJ, Harwood CR, et al. (2012) Cellular iron distribution in *Bacillus anthracis*. J Bacteriol 194: 932–940. doi: 10.1128/JB.06195-11 PMID: 22178968

79. Woodmansee AL, Imlay JA (2002) Quantitation of intracellular free iron by electron paramagnetic resonance spectroscopy. Meth Enzymol 349: 3–9. PMID: 11912920

80. Nakayashiki T, Inokuchi H (1997) Effects of starvation for heme on the synthesis of porphyrins in *Escherichia coli*. Mol Gen Genet 255: 376–381. PMID: 9267433
